# Marcksb plays a key role in the secretory pathway of zebrafish Bmp2b

**DOI:** 10.1371/journal.pgen.1008306

**Published:** 2019-09-23

**Authors:** Ding Ye, Xiaosi Wang, Changyong Wei, Mudan He, Houpeng Wang, Yanwu Wang, Zuoyan Zhu, Yonghua Sun

**Affiliations:** 1 State Key Laboratory of Freshwater Ecology and Biotechnology, Institute of Hydrobiology, Innovation Academy for Seed Design, Chinese Academy of Sciences, Wuhan, China; 2 College of Advanced Agricultural Sciences, University of Chinese Academy of Sciences, Beijing, China; 3 School of Basic Medical Sciences, Wuhan University, Wuhan, China; University of Pennsylvania School of Medicine, UNITED STATES

## Abstract

During vertebrate early embryogenesis, the ventral development is directed by the ventral-to-dorsal activity gradient of the bone morphogenetic protein (BMP) signaling. As secreted ligands, the extracellular traffic of BMP has been extensively studied. However, it remains poorly understood that how BMP ligands are secreted from BMP-producing cells. In this work, we show the dominant role of Marcksb controlling the secretory process of Bmp2b *via* interaction with Hsp70 *in vivo*. We firstly carefully characterized the role of Marcksb in promoting BMP signaling during dorsoventral axis formation through knockdown approach. We then showed that Marcksb cell autonomously regulates the trafficking of Bmp2b from producing cell to the extracellular space and both the total and the extracellular Bmp2b was decreased in Marcksb-deficient embryos. However, neither the zygotic mutant of *marcksb* (Z*marcksb*) nor the maternal zygotic mutant of *marcksb* (MZ*marcksb*) showed any defects of dorsalization. In contrast, the MZ*marcksb* embryos even showed increased BMP signaling activity as measured by expression of BMP targets, phosphorylated Smad1/5/9 levels and imaging of Bmp2b, suggesting that a phenomenon of “genetic over-compensation” arose. Finally, we revealed that the over-compensation effects of BMP signaling in MZ*marcksb* was achieved through a sequential up-regulation of MARCKS-family members Marcksa, Marcksl1a and Marcksl1b, and MARCKS-interacting protein Hsp70.3. We concluded that the Marcksb modulates BMP signaling through regulating the secretory pathway of Bmp2b.

## Author summary

Bone morphogenetic proteins (BMPs) are extracellular proteins which belong to the transforming growth factor-β (TGF-β) superfamily. BMP signaling is essential for embryonic development, organogenesis, and tissue regeneration and homeostasis, and tightly linked to various diseases and tumorigenesis. However, as secreted proteins, how BMPs are transported and secreted from BMP-producing cells remains poorly understood. In this study, we showed that Marcksb interacts with a molecular chaperon–Hsp70.3 to mediate the secretory pathway of BMP ligands during early development of zebrafish. Moreover, we discovered a novel phenomenon of “genetic over-compensation” in the genetic knock-out mutants of *marcksb*. To our knowledge, this is the first report that reveals the molecules and their related trafficking system mediating the secretion of BMPs. Considering the wide distribution of BMP and MARCKS within the human body, our work may shed light on the studies of BMPs secretion in organogenesis and adult tissue homeostasis. The finding of MARCKS in controlling BMP secretion may provide potential therapeutic targets for modulating the activity of BMP signaling and thus will be of interest to clinical research.

## Introduction

Early vertebrate development involves the formation and patterning of body plan, such as dorsoventral axis formation and anteroposterior axis formation. Bone morphogenetic protein (BMP) signaling gradient is critical for the specification of ventral and posterior cell fate [1]. Like other morphogens, the formation of BMP signaling gradient depends on several factors, including the graded transcription and secretion of BMP ligands, the extracellular transport of BMP ligands and the interaction between BMP ligands and their antagonists [2]. In zebrafish, the secreted ligands Bmp2b and Bmp7a act as heterodimers and bind to their receptors type I and type II to transduce signal and to phosphorylate the regulatory Smads (Smads 1, 5, and 9), which in turn regulate BMP target genes with Smad4 in the nuclei [3, 4].

As secreted ligands, the extracellular traffic of BMP homolog Dpp has been extensively studied in *Drosophila*. The long-range distribution of Dpp is mainly dependent on restricted extracellular diffusion [5], which process is regulated by glypican members of heparin sulfate proteoglycans [6]. In zebrafish, it was reported that BMP gradient is mainly determined by the graded expression of BMP ligands [7]. The secretion of several morphogens, such as WNTs, FGF-2 and Hedgehog has been studied in different animal models [8–11]. Recent study implies that the release of Dpp is regulated by inwardly rectifying potassium channel and calcium transients [12]. However, it remains poorly understood how the secretory pathway, including the intracellular trafficking and the secretion to extracellular space, of BMP ligands is regulated.

The myristoylated alanine-rich C-kinase substrate (MARCKS) is a ubiquitous substrate for protein kinase C (PKC). Two conserved domains within the MARCKS proteins are known to be critical for their functions: the N-terminal myristoylated domain helps anchoring MARCKS to the plasma membrane; and the phosphorylation site domain (PSD) domain serves as the site for MARCKS binding to actin filaments and calcium/calmodulin [13–17]. A notable function of MARCKS is to regulate the secretion of different substances including airway mucin [18, 19]. The well-studied regulated mucin secretion process via MARCKS involves its PKC and calcium/calmodulin dependent phosphorylation, high binding affinity with F-actin and membrane phosphoinositides, and interaction with intracellular molecular chaperons [20–23]. The MARCKS family proteins have also been reported to play various roles in gastrulation movements in *Xenopus* [24] and zebrafish [25], and the morphogenesis of neural tube in mouse [26] and chick [27]. However, the potential roles of MARCKS in morphogen secretion and embryonic patterning has never been studied and reported.

In this study, we unveiled a role of a MARCKS family member–Marcksb in dorsoventral patterning by regulating the BMP signaling activity through interacting with Heat-shock protein 70 (Hsp70) to control the secretion of BMP ligands. Interestingly, unlike the *marcksb* knockdown embryos showing dorsalization, the maternal-zygotic mutants of *marcksb* (MZ*marcksb*) showed mild ventralization, suggesting that genetic over-compensation arises in the MZ*marcksb* embryos. We further proved that the transcription of other MARCKS family members were strongly activated during oogenesis of MZ*marcksb* females, and Hsp70.3 –the MARCKS interaction protein was up-regulated at shield stage in MZ*marcksb* embryos, suggesting a sequential compensation of different genetic factors.

## Result

### Marcksb is required for specification of ventral cell fate

We previously identified zebrafish *marcksb* which is important for gastrulation movements [25]. To further understand the role of MARCKS family genes in early embryonic development, we examined the expression patterns of all the four members of MARCKS family–*marcksa*, *marcksb*, *marcksl1a* and *marcksl1b* during early embryogenesis. Among these four genes, *marcksb* is the only one showing maternal expression and is the most highly expressed one at the time of zygotic genome activation (S1 Fig).

**Fig 1:**
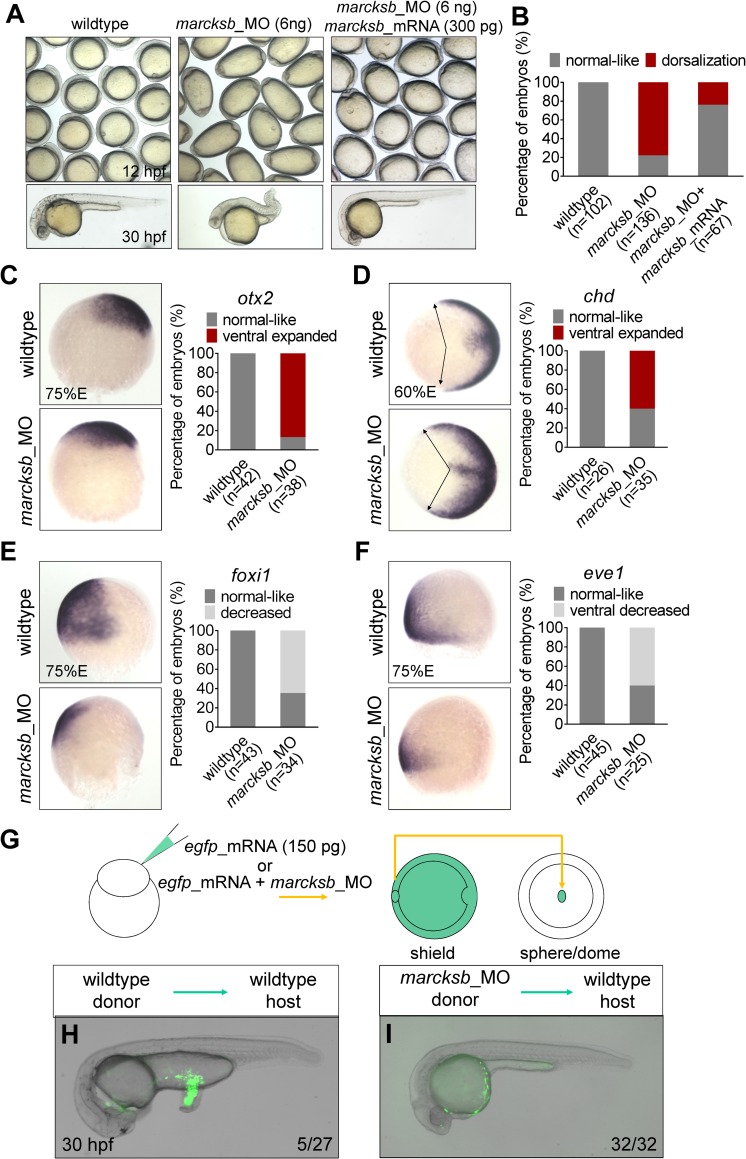
Marcksb is required for specification of ventral cell fate. (A) Knockdown of *marcksb* showed dorsalization defects, which could be rescued by overexpression of morpholino insensitive mRNA of *marcksb*. Up panels, field view of embryos at early-somite stage; lower panels, representative embryos at 24 hours post fertilization (hpf). (B) The percentage of embryos with normal-like or dorsalization defects. “n” represents the number of embryos we observed. (C-F) Whole-mount *in situ* hybridization (WISH) showed the expansion of dorsal markers *otx2* (neural ectoderm) (C) and *chordin* (dorsal margin) (D) expression, and the shrinkage of ventral markers *foxi1* (non-neural ectoderm) (E) and *eve1* (ventral margin) (F) expression in *marcksb* morphants. The percentage of embryos with indicated phenotype were shown aside their representative images. For *otx2*, *foxi1* and *eve1*, embryos are lateral view with animal-pole to the top and dorsal to the right; for *chd*, embryos are animal-pole view with dorsal to the right; arrows in *chd* panels indicate the expansion locations of signals. (G) A schematic showing the procedure of the tail organizer transplantation assay. (H) A representative wildtype-to-wildtype transplantation embryo showing an induction of ectopic tail by grafting the tail organizer region of the wildtype embryo. (I) A representative morphant-to-wildtype transplantation embryo without induction of ectopic tissue by grafting the tail organizer region of the *marcksb* morphant embryo. The ratio at the right corner indicates the number of embryos with the representative phenotype/the total number of observed embryos.

We then injected the morpholino (MO) blocking the translation of *marcksb* into zebrafish embryos and evaluated their phenotypes. The MO-injected embryos (morphants) showed spindle-like shape at bud stage (Fig 1A) and 77.9% showed dorsalization at 1 day post-fertilization (dpf) (Fig 1A and 1B). The defect of dorsalization in *marcksb* morphants was rescued by the injection of morpholino-insensitive *marcksb* mRNA (Fig 1A and 1B). Whole-mount *in situ* hybridization (WISH) analysis further confirmed the dorsalization defects in *marcksb* morphants, as revealed by the ventral expansion of *otx2* expression (labeling neural ectoderm) (Fig 1C) and *chordin* expression (labeling dorsal organizer) (Fig 1D). Accordingly, the expression level and region of ventral markers *foxi1* (labeling non-neural ectoderm) (Fig 1E) and *eve1* (labeling ventral margin) (Fig 1F) were strongly reduced.

To understand whether inhibition of *marcksb* could affect the development of ventral tissues, we performed a tail organizer graft assay as described previously (Fig 1G) [28]. We transplanted the wildtype ventral margin cells to the animal pole of wildtype host, and as expected, 5 out of 27 host embryos had extra tails (Fig 1H). In contrast, when the ventral margin cells of *marcksb* morphants were grafted to wildtype embryos, they failed to induce any extra tail structures (Fig 1I). Taken together, our data indicate that *marcksb* is required for the specification of ventral cell fate in zebrafish.

### Marcksb is required for activation of BMP signaling

**Fig 2:**
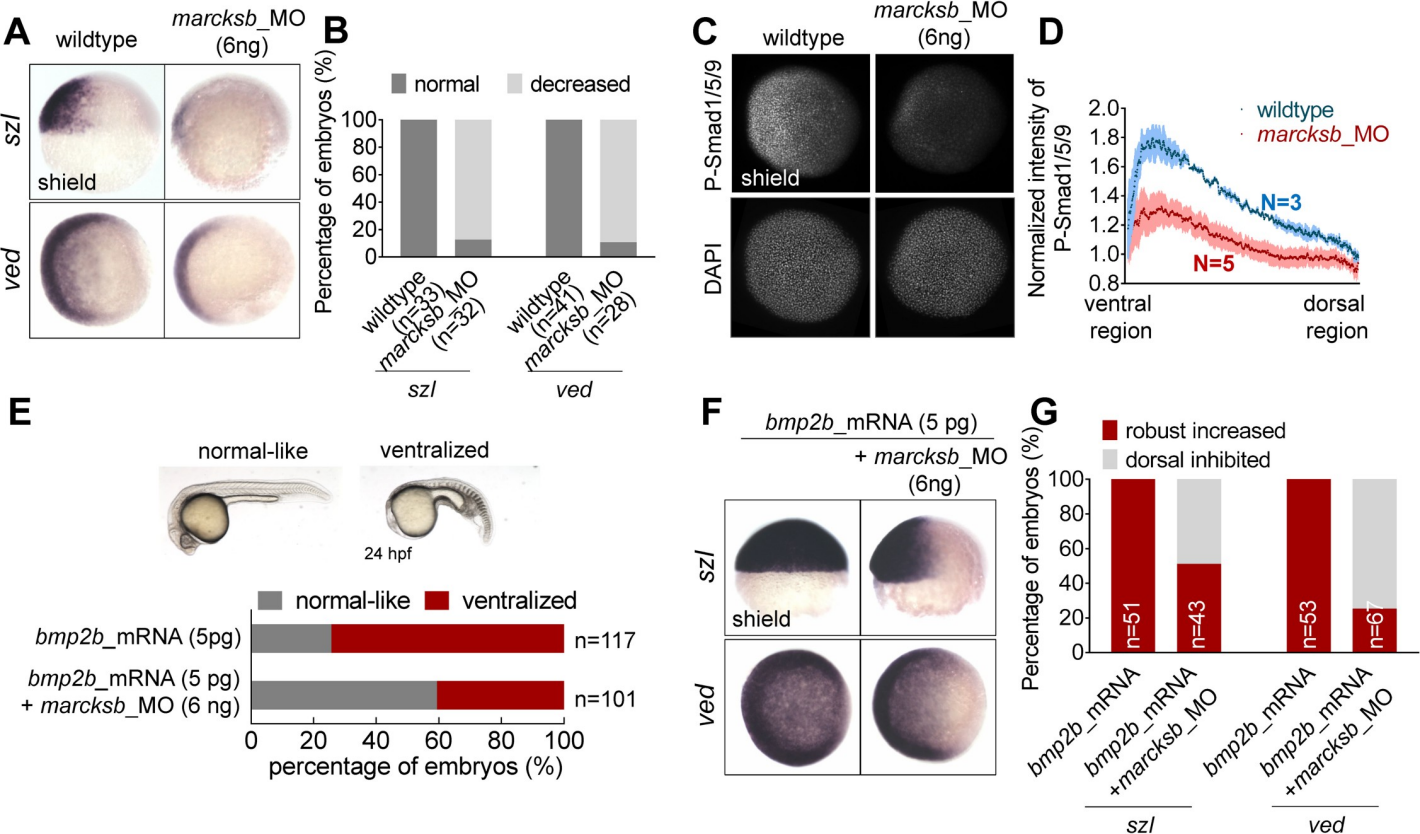
Marcksb is required for activation of BMP signaling. (A) WISH analysis showed that the transcriptional levels of downstream targets of BMP signaling—*szl* and *ved* were decreased in *marcksb* morphants. (B) The percentage of embryos with normal or decreased expression. “n” represents the number of embryos we observed. (C) The overall intensity of p-Smad1/5/9 at ventral region in *marcksb* morphants was dramatically decreased when compared with wildtype. (D) The normalized fluorescent intensity of P-Smad1/5/9. Error bars of light blue and light red show S.E.M. (E) Knockdown of *marcksb* could partially rescue the ventralization phenotype caused by injection of *bmp2b* mRNA (5 pg *bmp2b* mRNA per embryo). The statistical data are shown in the bar graphs with the number of observed embryos. “n” represents the number of embryos we observed. (F) Overexpression of *bmp2b* caused dramatic dorsal expansion of *szl* and *ved* expression while knockdown of *marcksb* in the *bmp2b*-overexpressed embryos partially restored their expression patterns. (G) The percentage of embryos with robust-increased or dorsal-inhibited expression. “n” represents the number of embryos we observed.

As zygotic BMP signaling plays a pivotal role in specifying the ventral cell fate, we next examined the BMP signaling activity in *marcksb*-depleted embryos. WISH showed that the expression of two direct transcriptional targets of BMP signaling—*szl* and *ved* were decreased in *marcksb* morphants compared to wildtype embryos (Fig 2A and 2B). We then performed immunofluorescence to measure the nuclei-enriched phosphorylation level of Smad1/5/9 (p-Smad1/5/9). The data showed that the relative intensity of p-Smad1/5/9 was lower in *marcksb* morphants than that in wildtype embryos (Fig 2C and 2D). Moreover, knockdown of *marcksb* could restore the ventralization phenotype in *bmp2b*-overexpressed embryos (5 pg of *bmp2b* mRNA per embryo) (Fig 2E). Accordingly, the injection of *bmp2b* caused robust expression expansion of *szl* and *ved* at dorsal region, and this dorsal expansion could be inhibited by knockdown of *marcksb* (Fig 2F and 2G). Altogether, our data indicate that *marcksb* knockdown leads to attenuation of BMP signaling and *marcksb* is required for the normal activation of BMP signaling.

### The activation of BMP signaling is related to phosphorylation and de-phosphorylation of Marcksb

**Fig 3:**
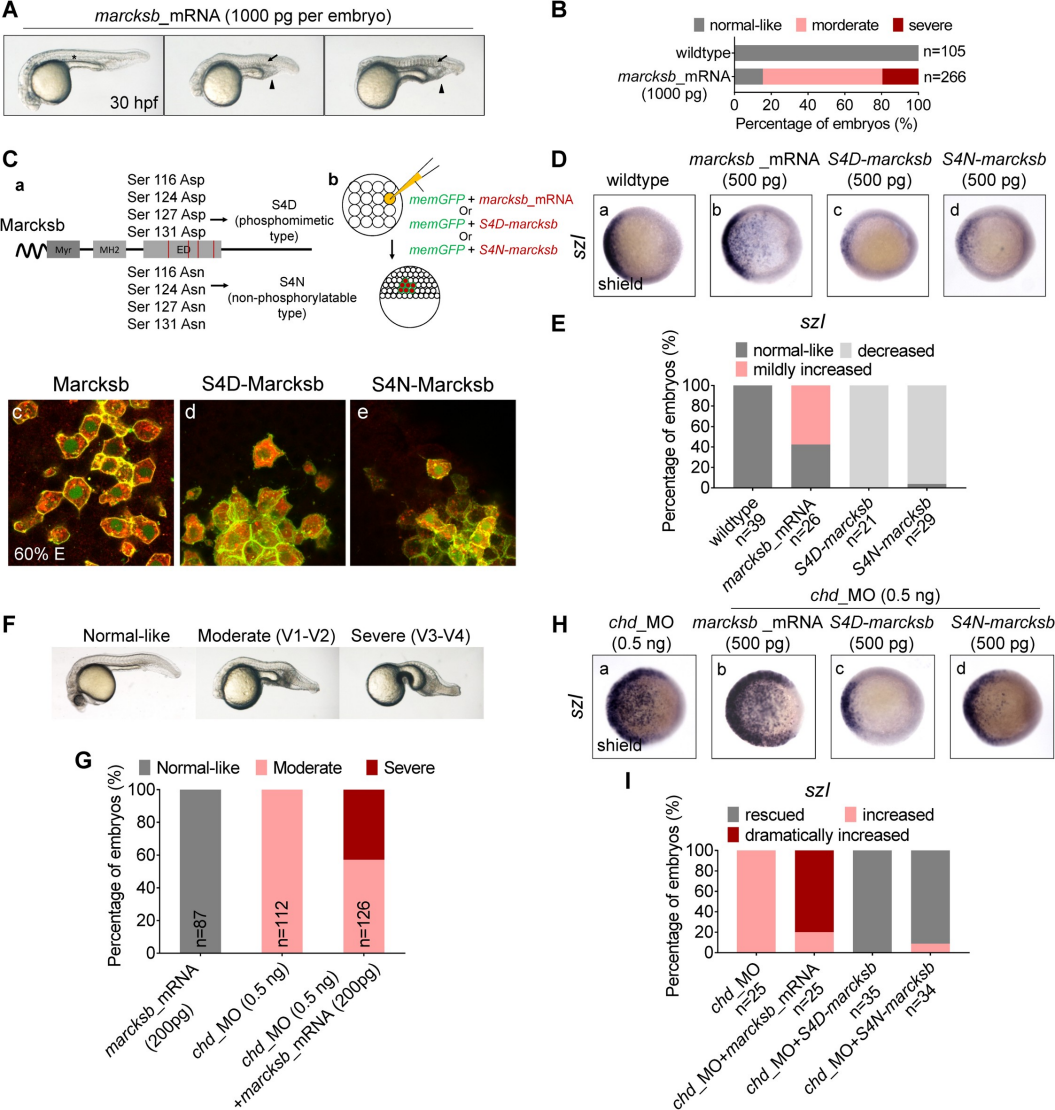
Phosphorylation and de-phosphorylation of Marcksb is required for the activation of BMP signaling. (A) Overexpression of *marcksb* at high dosage (1000 pg per embryo) caused ventralization defects. “asterisk” shows the notochord; “arrow” indicates the disappearance of notochord; “arrow head” indicates the enlarged blood island. (B) The percentage of embryos with normal-like, moderate ventralization or severe ventralization. “n” represents the number of embryos we observed. (C) Phosphorylation and de-phosphorylation mutation types of Marcksb altered the sub-cellular location of Marcksb. (C-a) a diagram showing mutated regions of S4D-Marcksb and S4N-Marcksb; (C-b) A schematic showing the procedure of mosaic overexpression assay; (C- c, d and e) confocal microscopy analysis at shield stage showed that wildtype Marcksb and S4N-Marcksb co-localized with memGFP while S4D-Marcksb did not. (D) Representative images showing the expression of *szl* in wildtype embryos (D-a), and the embryos injected with *marcksb* mRNA (D-b), *S4D-marcksb* mRNA (D-c) and *S4N-marcksb* mRNA (D-d) at shield stage. Embryos are animal-pole view with dorsal to the right. (E) The percentage of embryos with normal-like, decreased or mildly increased expression of *szl* in different experimental groups indicated in the (D). “n” represents the number of embryos we observed. (F, G) Genetic interaction was examined by co-injection of sub-dose *marcksb* mRNA (200 pg/embryo) and *chd*_MO. (F) representative embryos showing for phenotypes of normal-like, moderate ventralization (V1-V2, ventralization type 1 to type 2) and severe ventralization (V3-V4, ventralization type 3 to type 4); (G) The percentage of embryos with normal-like, moderate ventralization or severe ventralization. “n” represents the number of embryos we observed. (H) Representative images showing the expression of *szl* in the shield-stage embryos injected with *chd*_MO (H-a), *chd*_MO plus *marcksb* mRNA (H-b), *chd*_MO plus *S4D-marcksb* mRNA (H-c), and *chd*_MO plus *S4N-marcksb* (H-d). Embryos are animal-pole view with dorsal to the right. (I) The percentage of embryos with rescued, increased or dramatically increased expression of *szl* in different experimental groups indicated in the (H). “n” represents the number of embryos we observed.

We then conducted ectopic overexpression experiments of *marcksb*. Since *marcksb* was strongly maternally expressed (S1 Fig), injection of moderate dosage of *marcksb* mRNA (200pg per embryo) did not result in any visible effects, whereas injection of extremely high dosage of *marcksb* mRNA (1000 pg per embryo) led to ventralization (Fig 3A and 3B).

To test whether phosphorylation of Marcksb is required for the activation of BMP signaling, two mutated forms of Marcksb, the HA-tagged S4D-Marcksb (phosphomimetic type) and S4N-Marcksb-HA (non-phosphorylatable type) were generated according to previous study [29], and their mRNA were injected into one blastomere at 16-cell stage (Fig 3C-a, b). The wildtype Marcksb-HA mainly localized at the cell membrane (Fig 3C-c). In accordance with the notion that phosphorylation of Marcksb leads its translocation from the cell membrane to the cytoplasm [19], S4D-Marcksb mainly located inside cytoplasm (Fig 3C-d) and the S4N-Marcksb mainly co-localized with membrane-labeled EGFP (Fig 3C-e). We then examined *szl* expression in the embryos overexpressed with mutated *marcksb*. When compared with wildtype embryos, *marcksb* overexpressed embryos showed mildly increased expression of *szl* (Fig 3D-b), while both *S4D*-*marcksb* and *S4N-marcksb* overexpressed embryos showed decreased expression of *szl* (Fig 3D-c, d and 3E). These data suggest that both types of mutated Marcksb caused a dominant negative effect on regulating the BMP signaling activity.

To establish a sensitive way to examine the effects of *marcksb*-overexpression, we overexpressed *marcksb* in *chd_*MO injected embryos (*chd* morphants) in which BMP signaling was slightly enhanced. As expected, all the *chd* morphants showed moderate ventralization (Fig 3F and 3G). Strikingly, injection of moderate dosage of *marcksb* mRNA resulted in severe ventralization in *chd* morphants, although injection of the same dosage of *marcksb* mRNA did not result in any visible phenotype in wildtype embryos (Fig 3F and 3G). This phenomenon was further proved by WISH analysis of *szl* in those embryos at shield stage (Fig 3H-a, b and 3I). Strikingly, the elevated BMP signaling activity in *chd* morphants was dramatically inhibited by overexpression of both types of mutated Marcksb (Fig 3H-a, c, d and 3I). Thus, our data suggest that the phosphorylation and de-phosphorylation switch of Marcksb is tightly related to the activation of BMP signaling.

### Marcksb regulates BMP secretion cell autonomously

**Fig 4:**
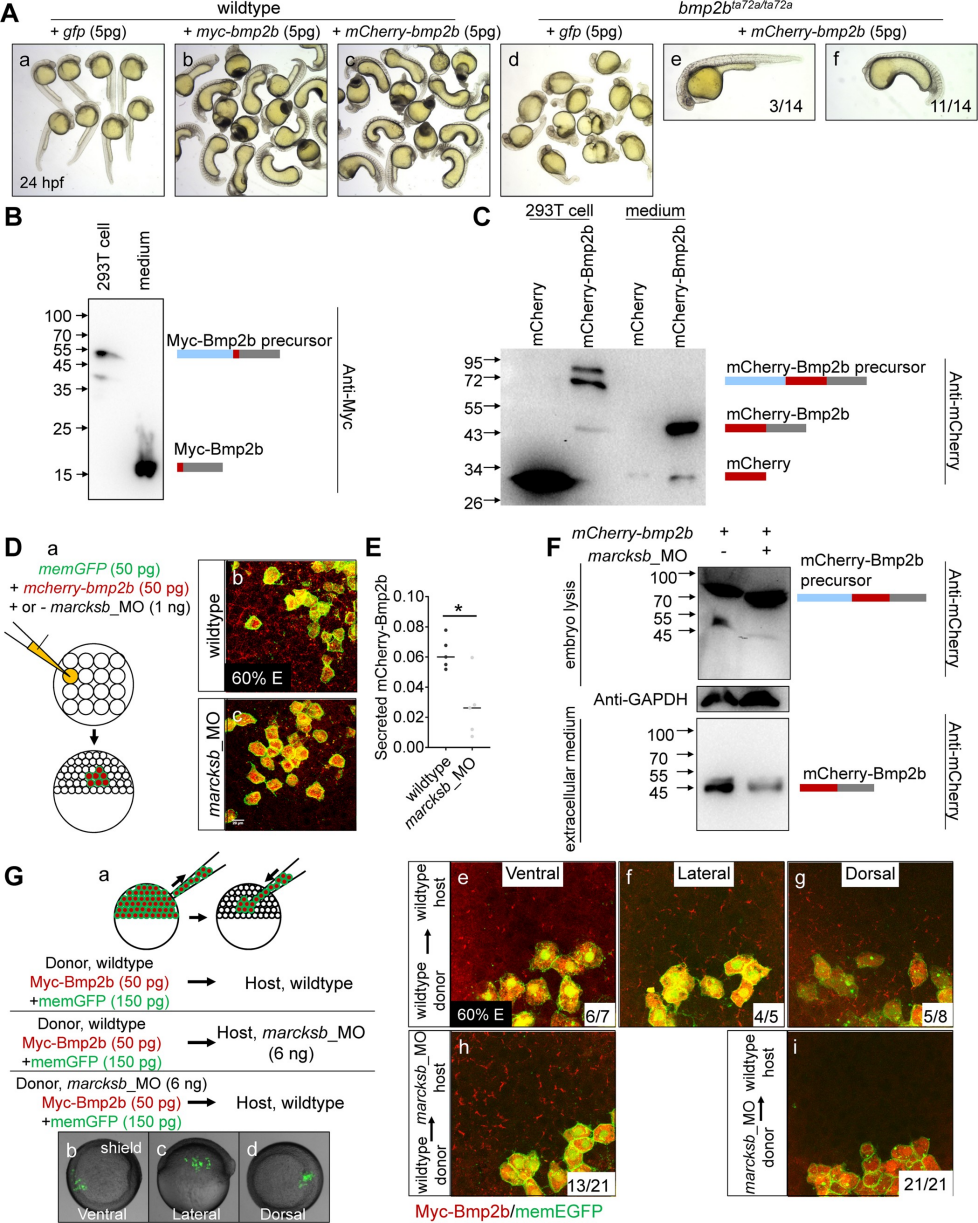
Marcksb cell autonomously regulates the extracellular level of Bmp2b. (A) Injection of either *myc-bmp2b* or *mcherry-bmp2b* caused severe ventralization at 24 hpf, and injection of *mcherry-bmp2b* could rescue the dorsalization of *bmp2b* mutant. (A-a) wildtype embryos; (A-b) *myc-bmp2b* injected embryos; (A-c) *mcherry-bmp2b* injected embryos; (A-d) *bmp2b* mutant (allele name: *bmp2b*^*ta72a/ta72a*^); (A-e) representative imaging showing *mcherry-bmp2b* rescued *bmp2b*^*ta72a/ta72a*^; (A-f) representative imaging showing ventralized *bmp2b*^*ta72a/ta72a*^ by over-dosage of *mcherry-bmp2b*. All embryos were at 24 hpf. (B) The pCS2-myc-bmp2b plasmid was transfected into 293T cells. Intracellular and extracellular Myc-Bmp2b were analyzed by immunoblotting using anti-Myc antibody. The positions of precursor and mature Bmp2b are illustrated schematically to the right of each gel. (C) The pCS2-mcherry-bmp2b plasmid and pCS2-mcherry plasmid were transfected into 293T cells separately. Intracellular and extracellular mCherry-Bmp2b or mCherry were analyzed by immunoblotting using anti-mCherry antibody. The positions of mCherry-Bmp2b precursor, mature mCherry-Bmp2b and mCherry alone are illustrated schematically to the right of each gel. (D) Knockdown of *marcksb* reduced the extracellular level of Bmp2b. (D-a) showed a diagram of mosaic injection; (D-b) showed the extracellular level of mCherry-Bmp2b in wildtype embryos; (D-c) showed the extracellular level of mCherry-Bmp2b was significantly reduced in the *marcksb* morphant embryos. (E) Quantitative measurement of secreted Bmp2b in wildtype and *marcksb* morphant embryos. The data were presented as scatter plots with median; *”: P < 0.01, from Student’s t-test. (F) The *mCherry-bmp2b* mRNA injected wildtype embryos and *marcksb* morphants were collected separately at shield stage. Total cell-derived and extracellular mCherry-Bmp2b were analyzed by immunoblotting using anti-mCherry antibody. The positions of mCherry-Bmp2b precursor and mature mCherry-Bmp2b are illustrated schematically to the right of each gel. (G) Assay on secreted Myc-Bmp2b of wildtype cells and *marcksb*-depleted cells by transplantation. (G-a) A diagram of cell transplantation; (G-b, c, d) The transplanted embryos at shield stage, indicating the location of transplanted cell populations: ventral (b), lateral (c) and dorsal (d); (G-e, f, g) When wildtype donor cells were transplanted into wildtype host, the extracellular Myc-Bmp2b were at a comparable level among ventral (e), lateral (f) and dorsal transplants (g); (G-h) When wildtype donor cells were transplanted into the *marcksb* morphants, the extracellular Myc-Bmp2b was not reduced; (G-i) When the *marcksb* morphants cells were transplanted into wildtype host, the extracellular Myc-Bmp2b was strongly inhibited.

Next, we asked whether Marcksb regulates the BMP signaling through the BMP secretory pathway. We first constructed tagged Bmp2b by insertion of mCherry or Myc tag right after the pro-domain of ligand protein according to previous study [30]. The overexpression of both *myc-bmp2b* and *mcherry-bmp2b* caused similar ventralization defect (Fig 4A a-c). To further confirm that fusion of mCherry to the N-terminal of Bmp2b does not interfere its *in vivo* function, we used the mCherry-bmp2b to rescue the mutant of *bmp2b* (*bmp2b*^*ta72a/ta72a*^). We did individual genotyping for embryos of *bmp2b*^*ta72a/ta72a*^ and *mcherry-bmp2b* injected *bmp2b*^*ta72a/ta72a*^. We found that all the *bmp2b*^*ta72a/ta72a*^ were dorsalized (Fig 4-d), while injection of *mcherry-bmp2b* mRNA could either rescue the dorsalization of *bmp2b*^*ta72a/ta72a*^ or cause ventralization (Fig 4A e-f). These data demonstrate that the insertion of myc- or mCherry-tag dose not interfere the biological function of Bmp2b.

To test whether Myc-Bmp2b or mCherry-Bmp2b can be properly cleaved and secreted, we transfected the plasmids containing either *myc-bmp2b* or *mcherry-bmp2b* and collected the cells and growth medium for immuno-analysis. For Myc-bmp2b, we found that the precursor (49 KD) was enriched in the cell lysis while the matured Myc-bmp2b (15 KD) in the medium (Fig 4B). For mCherry-Bmp2b, we transfected the cultured cells with plasmid containing mCherry alone as a control. Similarly, the precursor of mCherry-Bmp2b (74 KD) was mainly observed in the cell lysis while the matured mCherry-Bmp2b (41 KD) in the medium (Fig 4C). These data demonstrate that the insertion of Myc or mCherry does not interfere the proper cleavage and secretion of Bmp2b.

To investigate whether Marcksb regulates the secretion of Bmp2b, we then performed mosaic injection assay (Fig 4D-a). In the *mcherry-bmp2b* overexpressed embryos, the mCherry-Bmp2b could be detected outside the overexpressed-cells (Fig 4D-b and 4E). Strikingly, in the *marcksb* morphants, the level of mCherry-Bmp2b outside their producing cells was significantly decreased (Fig 4D-c and 4E). To further confirm that the above extracellular signal was from mature Bmp2b-mCherry, we detected the embryonic and extracellular mCherry-Bmp2b using immunoblotting. We revealed that there was mainly the precursor of mCherry-Bmp2b in the embryonic cells of wildtype or *marcksb* morphants and there were only properly cleaved matured mCherry-Bmp2b fusion proteins in the extracellular space, and the extracellular mCherry-Bmp2b was less in the *marcksb* morphants than that in wildtype embryos (Fig 4F). Moreover, it appeared that the total cleaved mature mCherry-Bmp2b of *marcksb* morphants was less and the precursor of mCherry-Bmp2b was more than that from wildtype embryos (Fig 4F). Thus, based on the above data, we conclude that *marcksb* is likely required for the intracellular trafficking and/or secretion of Bmp2b in which the cleavage of the Bmp2b precursor may be involved.

To investigate whether Marcksb regulated the secretion of Bmp2b in a cell-autonomous manner, we performed a transplantation assay. When the transplanted embryos developed to shield stage, we sorted the transplanted embryos of wildtype-to-wildtype into three groups according to the location of labeled descendants—ventral, lateral and dorsal (Fig 4G-a-d). Although the locations of labeled cells were different in those three groups, we did not observe any difference on the extracellular level of Bmp2b suggesting a similar capability of Bmp2b secretion from ventral to dorsal regions (Fig 4G-e-g). Subsequently, we transplanted the *myc-bmp2b-*overexpressed cells into the *marcksb* morphant host and found that they were capable of secreting Bmp2b in *marcksb* morphants as the myc-Bmp2b could be detected abundantly outside of the producing cells (62%, n = 21, Fig 4G-h). In contrast, the extracellular level of Bmp2b was significantly less in embryos with the *marcksb*-depleted cells transplanted to the wildtype host (100%, n = 21, Fig 4G-i). These data indicate that *marcksb* cell-autonomously regulates secretory pathway of Bmp2b.

### Maternal-zygotic mutants of *marcksb* (MZ*marcksb*) do not show visible dorsoventral defects

**Fig 5:**
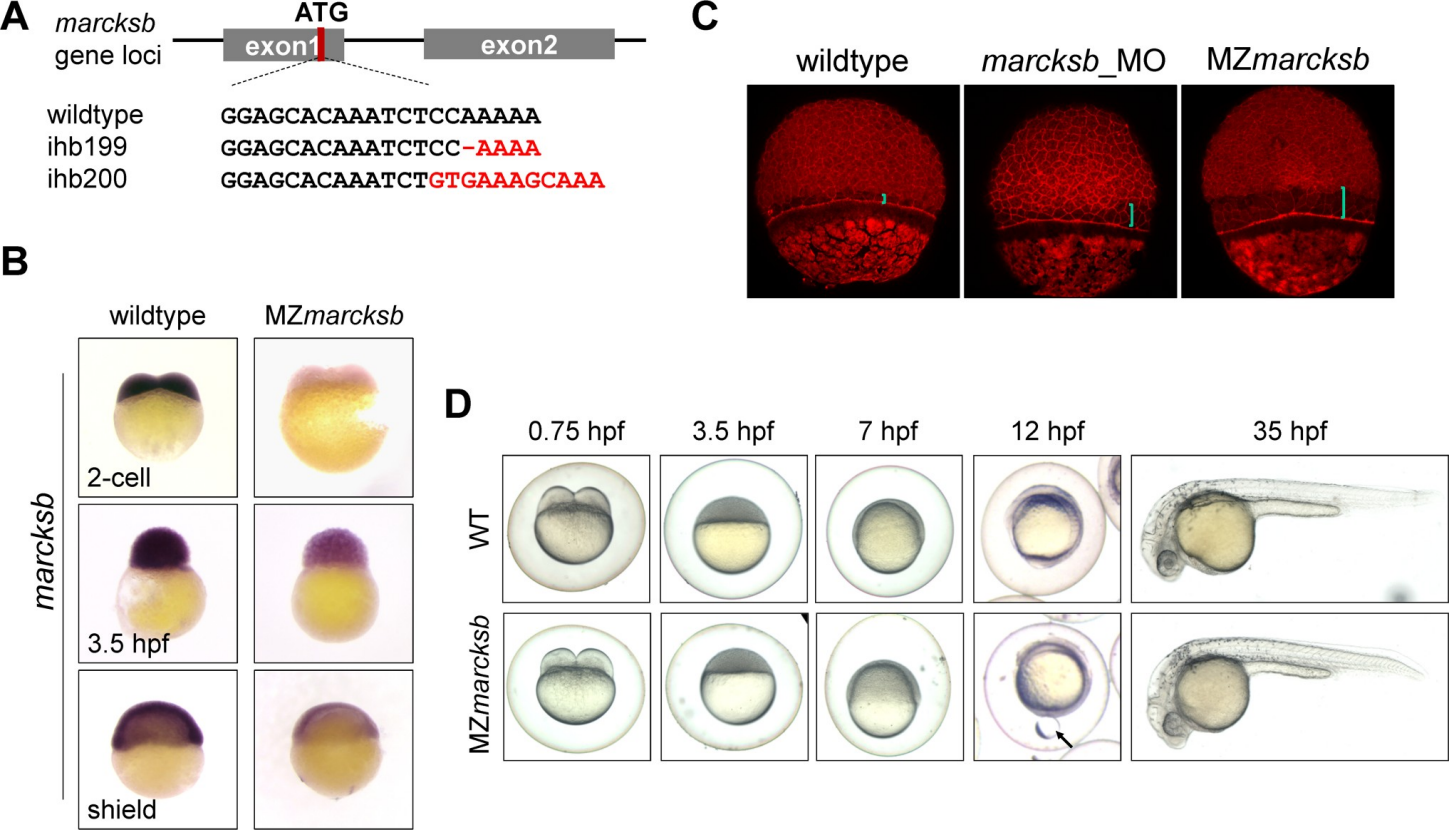
MZ*marcksb* do not show visible dorsoventral defects. (A) The diagram shows the gRNA target of *marcksb* genome locus and the genotypes of two individual *marcksb* mutants generated by CRISPR/Cas9. (B) The transcription level of *marcksb* was significantly decreased in MZ*marcksb* embryos. Embryos of 2-cell stage, high stage and shield stage were present. All the embryos were lateral view with animal-pole to the top. (C) Both MZ*marcksb* and *marcksb* morphants showed similar defect of epiboly movement. The blue bar indicates the distance between the inner layer of cells and the envelope layer. (D) The wildtype and MZ*marcksb* embryos at developmental stages of 0.75 hpf, 3.5 hpf, 7 hpf, 12 hpf and 35hpf. MZ*marcksb* showed yolk bulge phenotype during early somite stage but normal appearance of development at 35 hpf. “Arrow” indicates the extrusion of the vegetal-most part of the yolk.

To further unveil the role of *marcksb* on BMP signaling and dorsoventral patterning, we generated *marcksb* mutant by CRISPR/Cas9 mediated knockout (Fig 5A). After screening and verification by sequencing, we obtained two types of mutations–*marcksb*^*ihb199/ihb199*^ (https://zfin.org/ZDB-ALT-180302-14) and *marcksb*^*ihb200/ihb200*^ (https://zfin.org/ZDB-ALT-180302-15), both of which were predicted to shift their opening reading frames. There were no differences between these two alleles in phenotype analysis in subsequent studies. Therefore, we only presented the results of *marcksb*^*ihb199/ihb199*^ in the following part. To our surprise, the homozygous zygotic mutants (Z*marcksb*) did not show any early patterning defects and they could be raised up to adulthood, and we further generated maternal-zygotic mutant (MZ*marcksb*). WISH analysis showed that the expression of *marcksb* was dramatically decreased from 2-cell stage to shield stage in MZ*marcksb*, indicating that both maternal deposition and zygotic expression of *marcksb* were severely reduced in MZ*marcksb* (Fig 5B). This might be due to the failure of ribosome binding to mutated *marcksb* mRNA in the MZ*marcksb* embryos [31]. Surprisingly, MZ*marcksb* did not show any visible dorsoventral defects. However, we observed that the distance between the leading edges of enveloping layer (EVL) and deep cell layer (DCL) was enlarged in MZ*marcksb* during epiboly (Fig 5C). At bud stage, some MZ*marcksb* embryos showed a yolk bulge phenotype. A yolk droplet could be squeezed out of the body in some of the MZ*marcksb* embryos (Fig 5D arrow). These results indicated that MZ*marcksb* does not have dorsoventral defects but has moderate epiboly defects probably due to mild disorder of F-actin assembly [32].

### MZ*marcksb* shows elevated BMP signaling activity

**Fig 6:**
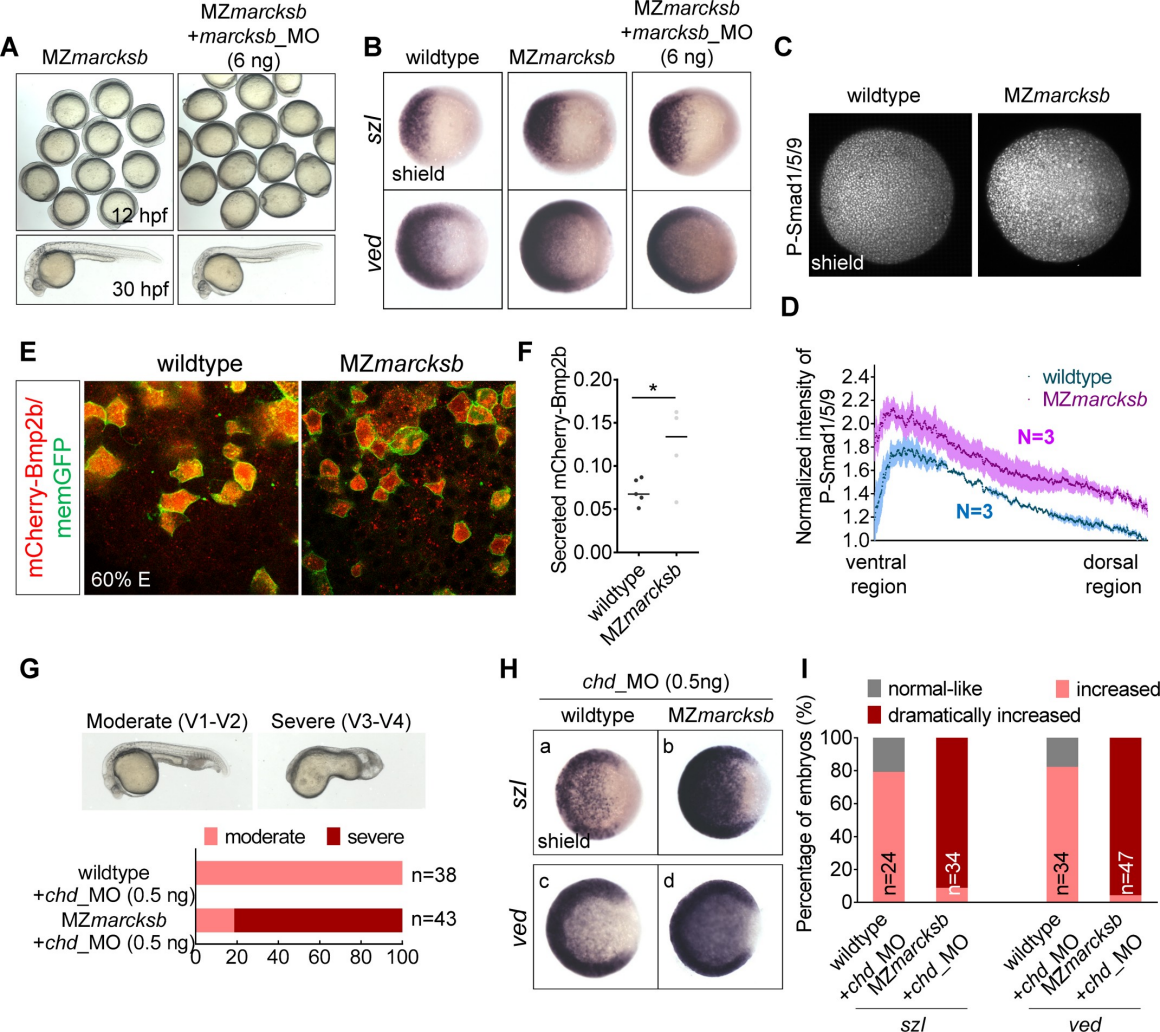
MZ*marcksb* showed genetic compensation on BMP signaling. (A, B) Injection of *marcksb*_MO in MZ*marcksb* did not cause defect of dorsalization. (A) morphological observation of MZ*marcksb* and *marcksb*_MO injected MZ*marcksb*. Up panels, field view of embryos at 12 hpf; lower panels, representative embryos at 30 hpf. (B) WISH analysis showed that the transcriptional levels of downstream targets of BMP signaling—*szl* and *ved* were at comparable level between MZ*marcksb* and *marcksb*_MO injected MZ*marcksb*. (C) The overall intensity of p-Smad1/5/9 at ventral region in MZ*marcksb* was slightly increased when compared with wildtype. (D) The normalized fluorescent intensity of P-Smad1/5/9. Error bars of light blue and light red show S.E.M. (E) The extracellular mCherry-Bmp2b was slightly increased in the MZ*marcksb* compared with wildtype embryos. (F) Quantitative measurement of secreted Bmp2b in wildtype and MZ*marcksb* embryos. The data were presented as scatter plots with median; *”: P < 0.01, from Student’s t-test. (G) Although knockdown of *chd* in wildtype embryos only resulted in moderate ventralization (V1-V2), knockdown of *chd* in MZ*marcksb* embryos resulted in severe ventralization (V3-V4). V1-V4, ventralization type 1 to type 4 embryos; the statistical data are shown in the bar graphs with the number of observed embryos indicated right. (H) After injection of *chd*_MO, the up-regulation of BMP signaling activity was much more robust in MZ*marcksb* than that in wildtype embryos shown by WISH analysis of *szl* (a vs b) and *ved* (c vs d). The embryos are at shield stage and animal-pole view with dorsal to the right; (I) The percentage of embryos with normal-like, increased, and dramatically-increased expression of *szl* and *ved*. “n” represents the number of embryos we observed.

As MZ*marcksb* did not show any dorsoventral defects as *marcksb* morphants, we speculated that there was genetic compensation occurring in the MZ*marcksb* [33, 34]. To challenge the hypothesis, we first injected the *marcksb*_MO into the MZ*marcksb*. The injected MZ*marcksb* showed to be *marcksb*_MO resistant and has no obvious dorsalization defect (Fig 6A). WISH analysis also showed that the expression of BMP targets—*szl* and *ved* (Fig 6B) and dorsal and ventral ectoderm markers—*otx2* (S2A and S2B Fig) and *foxi1* (S2E and S2F Fig) did not show any obvious difference between the MZ*marcksb* embryos with or without *marcksb*_MO injection. All these indicate that MZ*marcksb* is a null mutant of *marcksb* which does not respond to *marcksb*_MO and the *marcksb* morphant phenotype in wildtype embryos is a specific effect.

However, when we carefully compared the expression of *szl* and *ved* in wildtype and MZ*marcksb* embryos, we found a slight increase of expression levels of *szl* and *ved* in the MZ*marcksb* embryos, suggesting an elevation of BMP signaling activity in MZ*marcksb*. To further confirm this finding, we detected and compared the nuclear localization of P-Smad1/5/9 in MZ*marcksb* and wildtype embryos at shield stage. Consistent with the WISH results, the intensity of P-Smad1/5/9 was significantly increased in MZ*marcksb* (Fig 6C and 6D). Additionally, we performed the live imaging of Bmp2b by mosaic injection of *mcherry-bmp2b* mRNA. We found higher amount of mCherry-Bmp2b outside their producing cells in MZ*marcksb* in comparison with that in wildtype embryos (Fig 6E and 6F). Finally, we found that the *chd*_MO injection only led to moderate ventralization phenotype (V1-V2) in wildtype embryos, but it resulted in very severe ventralization (V3-V4) in MZ*marcksb* embryos (Fig 6G). Consistently, WISH analysis showed that knockdown of *chd* caused more robust increase of *szl* and *ved* expression in MZ*marcksb* than those in the wildtype embryos (Fig 6H and 6I). Taken together, these data strongly suggest that genetic compensation occurred in the MZ*marcksb* embryos, and moreover, the BMP signaling activity was even “over-compensated”.

### Up-regulation of the MARCKS family members and its interaction protein Hsp70.3 over-compensates the genetic loss of *marcksb*

To better understand the compensation network in MZ*marcksb*, we carried out RNA-Seq analysis of the MZ*marcksb* mutant at shield stage (S1 Dataset). Consistent with WISH analysis of *marcksb* (Fig 5B), RNA-Seq data showed that the expression level of *marcksb* was significantly reduced in MZ*marcksb* (Table 1). We also found that *bmp7a* was up-regulated in MZ*marcksb*, which is consistent to our observation that BMP signaling activity was slightly enhanced in MZ*marcksb* embryos (Table 1), as *bmp7a* is a transcriptional target of the BMP signaling [35, 36]. To dig out the main compensation factors, we searched for the list of differentially expressed genes (S1 Dataset) and found that *hsp70*.*3* was the second most up-regulated gene after *hsp90aa1*.*2* on the list of up-regulated genes in MZ*marcksb*.

**Table 1:** Selected genes were listed as below with average expression levels (represented by Reads Per Kilobase per Million mapped reads (RPKM)), log2foldchange and adjusted P value (P^adj^) from RNA-seq analysis of wildtype and MZ*marcksb* embryos at shield stage.

Gene name	Ensembl ID	wildtype	MZ*marcksb*	log_2_^FoldChange^	p^adj^
		FPKM	FPKM		
***hsp70*.*3***	ENSDARG00000021924	3.0	271.0	4.5	4.5E-41
***bmp7a***	ENSDARG00000018260	409.5	704.3	0.7	8.7E-04
***marcksa***	ENSDARG00000004049	126.4	144.4	0.2	7.9E-01
***marcksl1a***	ENSDARG00000039034	1312.7	1468.3	0.2	6.4E-01
***marcksl1b***	ENSDARG00000035715	8007.3	7940.5	0.0	9.8E-01
***marcksb***	ENSDARG00000008803	5242.5	760.1	-2.7	6.4E-75

**Fig 7:**
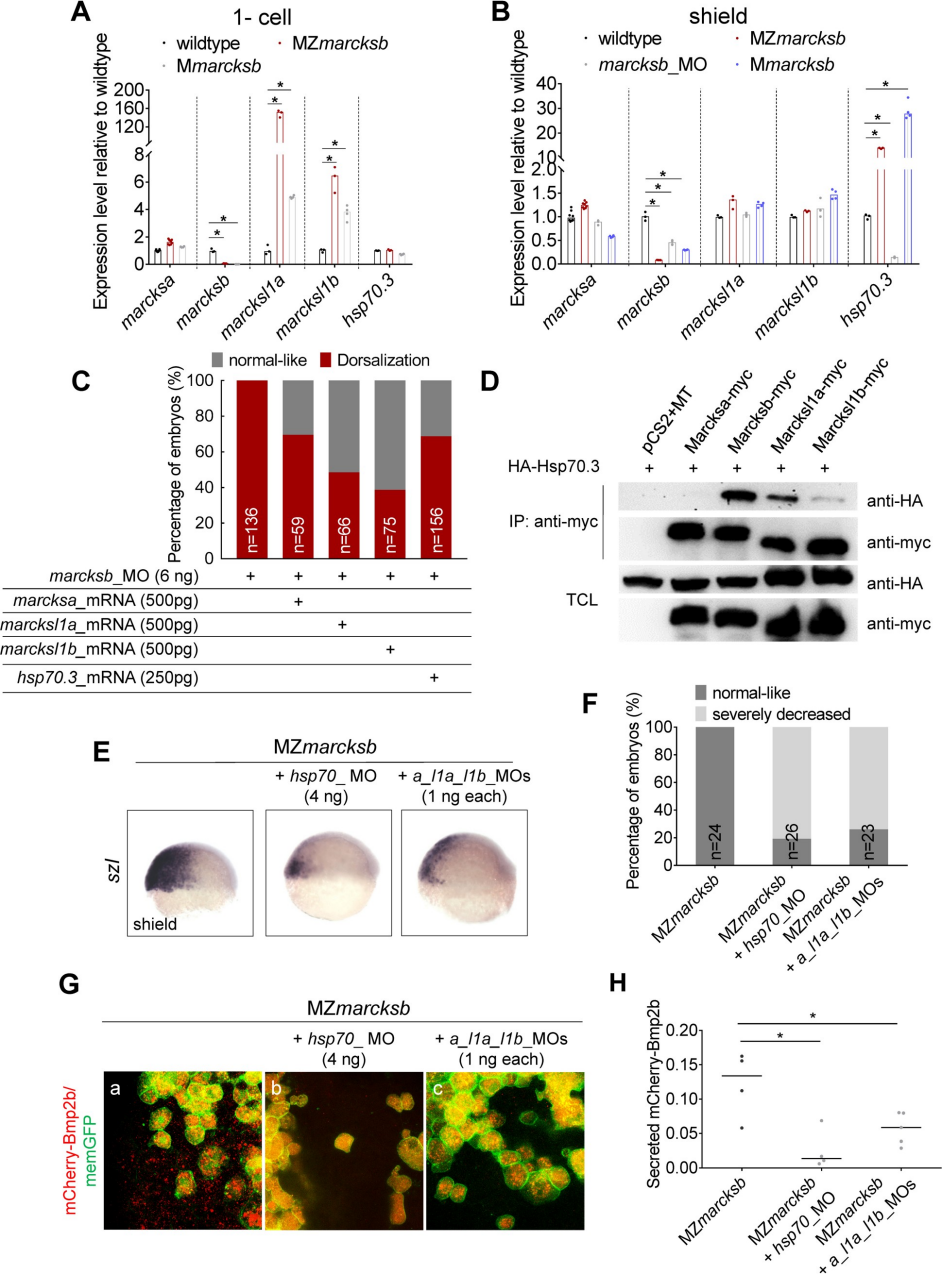
Up-regulation of the MARCKS family members and its interaction protein Hsp70.3 over-compensates the genetic loss of *marcksb*. (A, B) RT-qPCR analysis on the expression of *marcksa*, *marcksb*, *marcksl1a*, *marcksl1b* and *hsp70*.*3* in the embryos indicated in the Fig at 1-cell stage (A) and shield stage (B). The data were presented as scatter plots with bar representing median value relative to respective transcript levels measured in wildtype embryos. “*”: P < 0.01, from Student’s t-test. (C) Overexpression of *marcksa*, *marcksl1a*, *marcksl1b* or *hsp70*.*3* partially rescued the dorsalization defects in *marcksb* morphants. “n” represents the number of embryos we observed. (D) Co-immunoprecipitation revealed that Hsp70.3 had high binding affinity with Marcksb and moderate binding affinity with Marcksl11a and Marcksl1b, but relative low binding affinity with Marcksa. TCL: total cell lysis. The molecular mass of Marcks-myc is around 70KD. (E) The expression of *szl* was remarkably reduced in MZ*marcksb* injected with either *hsp70*_MO or *marcksa_l1a_l1b*_MO. The embryos are at shield stage and lateral view with dorsal to the right. (F) The percentage of embryos with normal-like and severely decreased expression of *szl*. “n” represents the number of embryos we observed. (G) Hsp70 interacts with Marcksa, Marcksl1a and Marcksl1b to maintain sufficient level of extracellular Bmp2b in MZ*marcksb*. (a-c) Compared with MZ*marcksb* embryos (a), knockdown of either *hsp70* (b) or a combination of *marcksa*, *marcksl1a* and *marcksl1b* (c) remarkably reduced the level of extracellular Bmp2b in MZ*marcksb*. (H) Quantitative measurement of secreted Bmp2b in embryos of MZ*marcksb*, MZ*marcksb* injected with either *hsp70*_MO or *marcksa_l1a_l1b*_MO. The data were presented as scatter plots with median; “*”: P < 0.01, from Student’s t-test.

We then performed RT-qPCR analysis of all the MARCKS genes and the *hsp70*.*3* in MZ*marcksb*, maternal mutant of *marcksb* (M*marcksb*), *marcksb* morphants and wildtype embryos. Interestingly, in MZ*marcksb*, all the other MARCKS members, *marcksa*, *marcksl1a* and *marcksl1b* were all significantly up-regulated at 1-cell stage (Fig 7A), but not at shield stage (Fig 7B). Moreover, *hsp70*.*3* was up-regulated in MZ*marcksb* at shield stage but not 1-cell stage (Fig 7A and 7B). These data suggest a phenomenon of sequential genetic response by MARCKS family members and *hsp70*.*3* to maternal-zygotic loss of *marcksb* from oogenesis to early embryogenesis. Interestingly, this phenomenon could also be seen in the M*marcksb* embryos, suggesting that the genetic responses is independent of zygotic activation of *marcksb* in M*marcksb* (Fig 7A and 7B). Unlike the upregulation of *hsp70*.*3* in MZ*marcksb* or M*marcksb* embryos, the expression of *hsp70*.*3* was significantly decreased in *marcksb* morphants. To further confirm whether this genetic compensation persists even after wildtype zygotic gene activation of *marcksb*, we knocked down *marcksb* in M*marcksb* embryos and found that those embryos did not show any dorsalization defect (S2C, S2D, S2G, S2H and S2I–S2L Fig). Together, these results suggest that the MARCKS family members and *hsp70*.*3* were up-regulated sequentially from oogenesis to early embryogenesis to response to the genetic loss of *marcksb*, and these genetic responses appear to be independent of zygotic activation of *marcksb*.

We then asked whether the other three MARCKS family members or *hsp70*.*3* could compensate the function of *marcksb* in the absence of functional Marcksb, we injected *marcksa*, *marcksl1a*, *marcksl1b* or *hsp70*.*3* mRNAs individually into *marcksb* morphants and we found that all of them could partially rescue the dorsalization defect of *marcksb* morphants (Fig 7C). These results suggest that the other MARCKS family members have the potential to replace the role of the Marcksb in the MZ*marcksb* mutant and *hsp70*.*3* may have some genetic interaction with MARCKS family genes in regulating BMP signaling activity.

As it was reported previously that MARCKS interacts with HSP70 to regulate mucin secretion in human airway epithelial cells [37], We performed *in vitro* co-IP analysis to test whether the zebrafish MARCKS also bind to Hsp70.3. The Hsp70.3 had the highest binding affinity to Marcksb and moderate binding affinity to Marcksl11a and Marcksl1b. However, the binding affinity between Hsp70.3 and Marcksa is rather weak (Fig 7D), which is in consistent to the relatively low rescue efficiency of *marcksa*-overexpression in *marcksb* morphants (Fig 7C).

To further address whether *hsp70*.*3*, *marcksa*, *marcksl1a* and *marcksl1b* over-compensate the BMP signaling activity in MZ*marcksb* embryos, we performed loss-of-function analysis of those genes in MZ*marcksb*. We found that the expression of *szl* was severely decreased in MZ*marcksb* injected with moderate dosage of *hsp70*_MO (previously published morpholinos against all three variant splicing isoforms [38]) or a combination of morpholinos against *marcksa*, *marcksl1a* and *marcksl1b* (previously published morpholinos, for abbreviation, *a_l1a_l1b*_MOs [39, 40]) (Fig 7E and 7F), while the same dosage of *hsp70*_MO or *a_l1a_l1b*_MOs only led to slightly decreased *szl* expression in wildtype embryos (S3 Fig). To further verify the compensatory role of Hsp70.3 and other MARCKS members in MZ*marcksb*, we performed the experiments with CRISPR/Cas9 knockout method using the gRNAs against *hsp70*, *marcksa*, *marcksl1a* and *marcksl1b*. All the gRNAs were validated by sequencing of the target sites (S4 Fig). We found that the expressions of *szl* and *ved* were severely decreased in MZ*marcksb* embryos injected with either *hsp70*_gRNA or a mixer of MARCKS gRNAs (S5 Fig), which was similar to the observations from their MOs mediated knockdown in MZ*marcksb*. All these data revealed that *hsp70*.*3*, *marcksa*, *marcksl1a* and *marcksl1b* over-compensated the BMP signaling activity in MZ*marcksb* embryos.

We then performed BMP imaging in MZ*marcksb* using mCherry-fused Bmp2b as reporter. Although we previously observed a higher level of extracellular Bmp2b in the MZ*marcksb* than that in the wildtype embryos (Fig 6E and 6F), knockdown of either *hsp70* or a combination of *marcksa*, *marcksl1a* and *marcksl1b* remarkably reduced the secreted Bmp2b level in MZ*marcksb* (Fig 7G and 7H). These lines of evidence demonstrated that the genetic over-compensation was due to the cooperation between the other members of MARCKS family and the molecular chaperone–Hsp70.3, which might even lead to mildly enhanced Bmp2b secretion level and BMP signaling activity in MZ*marcksb* embryos.

### Marcksb interacts with Hsp70.3 to regulate the secretory pathway of Bmp2b during dorsoventral patterning

**Fig 8:**
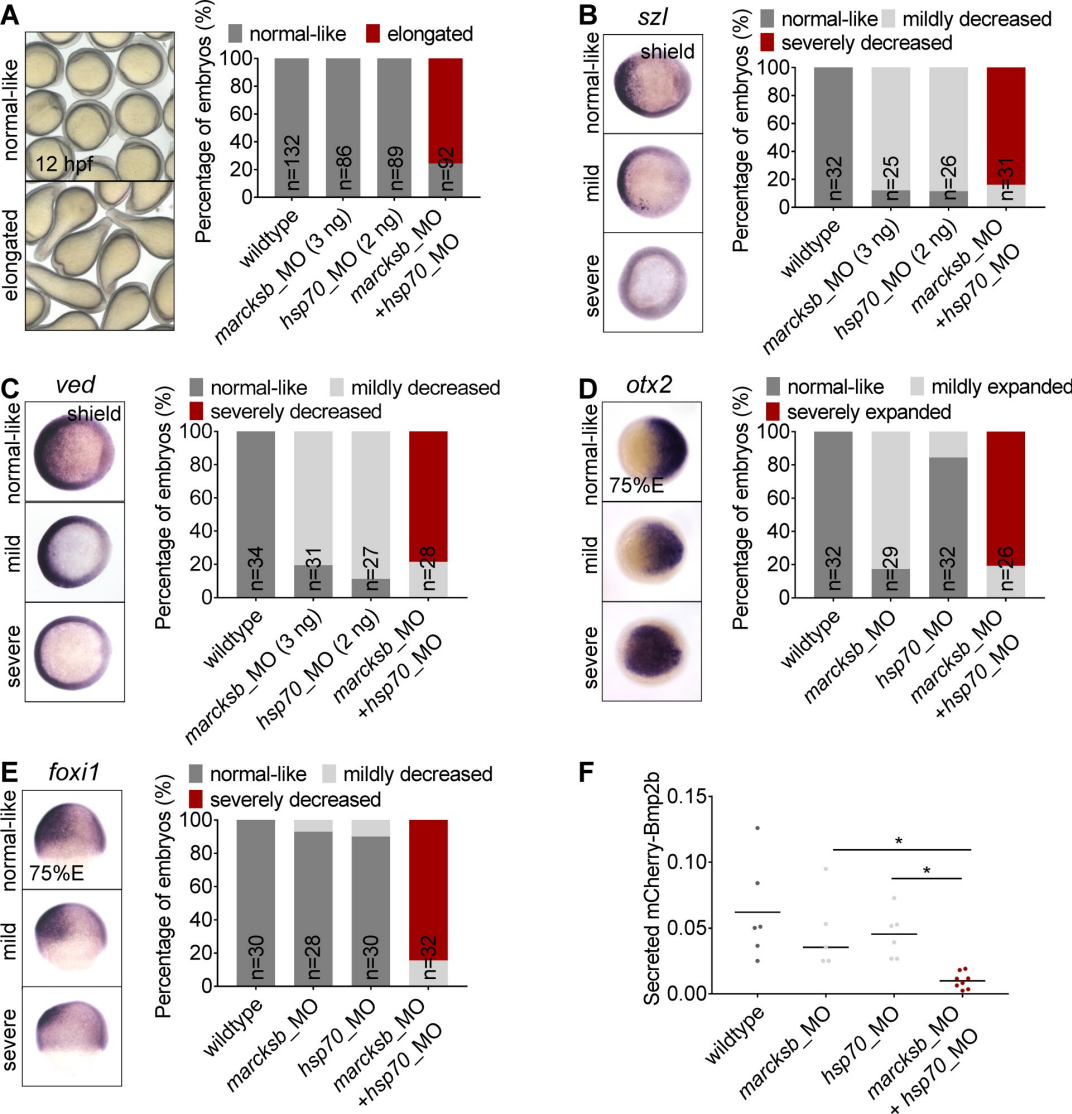
Marcksb interacts with Hsp70.3 to regulate the dorsoventral patterning and the extracellular level of Bmp2b. (A) Morphological phenotypes of embryos at 12 hpf, injected with sub-dosages of morpholinos against *marcksb* or *hsp70* or the combination of both (*marcksb*_MO: 3 ng/embryo; *hsp70*_MO: 2 ng/embryo). (B-E) WISH analysis of *szl* (B), *ved* (C), *otx2* (D), *foxi1* (E). The percentage of embryos with different phenotypes for each group were indicated in the graph. Dosages of morpholino per embryo are indicated in the figure panel. For *szl*, *ved* and *otx2*, embryos are animal-pole view with dorsal to the right; for *foxi1*, embryos are lateral view with dorsal to the right. “n” represents the number of embryos we observed. (F) Quantitative measurement of secreted Bmp2b. The data were presented as scatter plots with median; “*”: P < 0.01, from Student’s t-test.

**Fig 9:**
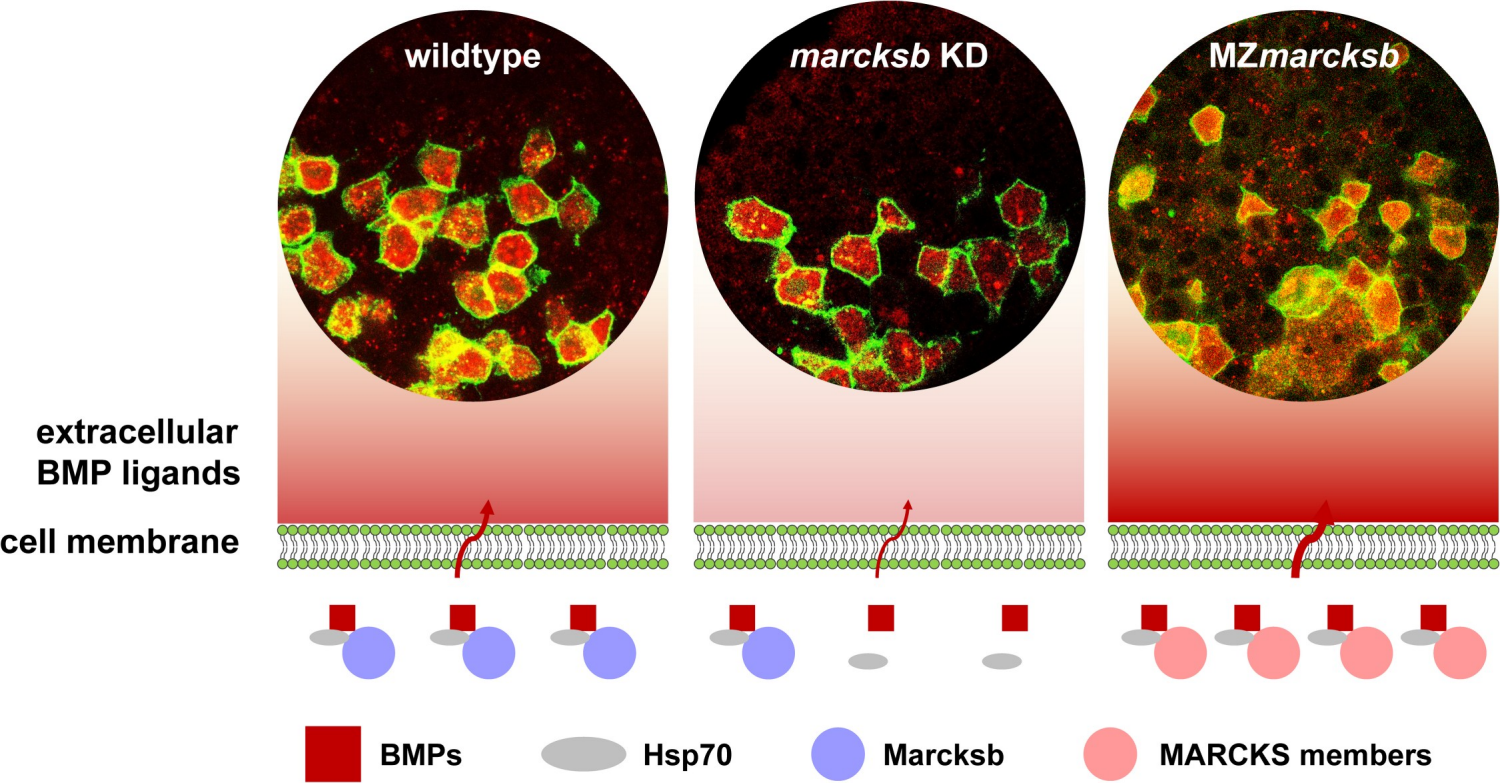
A model shows MARCKS-mediated BMPs secretory process in wildtype embryos, *marcksb* morphants and MZ*marcksb* embryos. A model shows MARCKS-mediated BMPs secretory process in the cells of wildtype embryos, *marcksb* morphants (*marcksb* KD) and MZ*marcksb* embryos. In wildtype embryo, Marcksb dominantly interacts with Hsp70 to mediate the secretion of BMPs; in *marcksb* morphant embryos, reduction of Marcksb proteins interfere the secretion of BMPs; in MZ*marcksb* embryos, *marcksa*, *marcksl1a* and *marcksl1b* (MARCKS members) are up-regulated maternally and *hsp70*.*3* is up-regulated zygotically, which leads to a massive production of functional MARCKS-Hsp70 complex and increased extracellular level of BMPs.

To investigate whether Marcksb interacted with Hsp70.3 to regulate the secretory pathway of Bmp2b in wildtype embryos, we performed a series of genetic interaction experiments. Firstly, we knocked down *hsp70* by injection of full dosage of *hsp70*_MO. We found that knockdown of *hsp70* led to inhibition of BMP signaling activity shown by decreased expression of *szl* and *ved*, which could be partially rescued by morpholino-resistant mRNA injection (S6 Fig). In the embryos co-injected with sub-dosage of *marcksb*_MO and *hsp70*_MO, a series of criteria were performed for careful evaluation: spindle shape of morphological defect was visible at early-somite stage (Fig 8A); the expression of BMP targets *szl* and *ved* was dramatically decreased (Fig 8B and 8C); the expression of neuronal dorsal marker *otx2* was expanded to the ventral region (Fig 8D); the expression of epidermal marker *foxi1* was decreased (Fig 8E). By contrast, in the embryos injected with either *marcksb*_MO or *hsp70*_MO alone did not show such defects (Fig 8A–8E). The Bmp2b live imaging was performed by transplantation of wildtype cells or cells injected with sub-dosage of *hsp70*_MO or *marcksb_*MO either alone or together into wildtype host. To our expectation, there were very few signals of the mCherry-Bmp2b outside the source cells in the *hsp70* and *marcksb* double morphants, unlike that the mCherry-Bmp2b could be efficiently secreted from the source cells in the wildtype, or the embryos injected with sub-dosages of *hsp70_*MO or *marcksb_*MO (Fig 8F). All these data demonstrate that Marcksb interacts with Hsp70.3 to regulate the secretory process of Bmp2b in wildtype embryos, and BMP signaling activity is over-compensated in MZ*marcksb* embryos likely by mildly enhanced secretory pathway involving MARCKS family members and Hsp70.3 (Fig 9).

## Discussion

Bmp2b acts as a major morphogen to specify ventral cell fate during early embryogenesis. In this study, we found that the secretory pathway of Bmp2b requires a MARCKS family member-Marcksb and its interaction protein Hsp70.3. Interestingly, we revealed that a phenomenon of genetic over-compensation, which has seldomly described in previous studies, happened in the MZ*marcksb*, which was achieved by sequential up-regulation of the other MARCKS family members and Hsp70.3.

### The possible roles of MARCKS in the secretory pathway of Bmp2b

MARCKS is known to be involved in regulating secretion of many proteins in various cell types. The role of MARCKS in mucin secretion in the airway has been intensively studied [19, 22, 37, 41–46]. The translocation of MARCKS from the cell membrane to the cytoplasm upon phosphorylation by PKCδ is the initial step allowing MARCKS binding to the mucin granules [44], and this binding requires the interaction among translocated MARCKS, Hsp70 and Cysteine string protein (CSP) [22, 37]. After dephosphorylated by protein phosphatase I and 2A, MARCKS mediates the mucin granules binding to the myosin V and move along the cytoskeleton to the cell membrane [20]. In our study, the interaction between MARCKS and Hsp70 and the Phosphorylation of Marcksb both affect the extracellular level of Bmp2b, which indicates that MARCKS acts similarly to its role in mucin secretion in the intracellular trafficking and the secretion of Bmp2b.

The maturation of TGF-β superfamily ligands, such as BMPs, requires endoproteolytic cleavage of the prodomain from BMPs precursors (ProBMPs) which coincides with the intracellular trafficking process [47, 48]. Our data show that Bmp2b is properly cleaved before it being secreted to the extracellular space, as only properly cleaved Bmp2b is detected in the extracellular medium. The extracellular level of Bmp2b is much lower in *marcksb*-deficient embryo, indicating that *marcksb* is required for the secretory pathway of BMP ligands. Besides, we also noticed that the proBmp2b level was slightly increased and the cleaved Bmp2b level was slightly decreased in the embryonic lysis of *marcksb*-deficient embryo when compared with wildtype embryo. In consideration of the key role of MARCKS in intracellular trafficking system, we propose that proBmp2b would not traffic properly to the place where it is cleaved without the help of MARCKS. Moreover, the defective cleavage of proBMPs may further interfere dimerization, folding, and secretion of the active ligands [49, 50]. Therefore, it is possible that *marcksb* and *hsp70* are required for one or several steps in the whole secretory pathway of BMPs, which mainly includes the intracellular trafficking along with endoproteolytic cleavage and the secretion to extracellular space.

### MARCKS regulates cell fate determination independent of cell migration

Embryonic gastrulation includes dynamic events of cell migration and cell fate determination, both of which some molecules are involved in. One example is that the ventral to dorsal BMP signaling gradient transducing through Alk8 and Smad5 can create loose cell-cell adhesiveness at ventral region and allow ventral cells migrating dorsally [51]. This effect of BMP signal is different from its classical role in ventral cell fate determination and possibly is achieved by transcriptional activation of gene regulating cadherin function [51]. Our study provides another example on how one molecule could act on both morphogenesis and cell fate determination. It is widely accepted that MARCKS is required for gastrulation movements, which might be related to its binding with phosphoinositides [52] and F-actin [24]. Although the previous MARCKS knockdown studies in *Xenopus* and zebrafish mainly focused on its function on gastrulation movements [24, 25], they could not exclude the possibility that MARCKS family members also participate in embryonic patterning before or during gastrulation. In the present study, we also observed epiboly defects in both *marcksb* morphants and *marcksb* mutants, which is consistent to its classical role in regulation of cell migration. For the first time, however, we revealed that zebrafish *marcksb* is also required for dorsoventral patterning, and the function is achieved by interacting with Hsp70.3 to regulate the secretory process of BMPs, a type of morphogen crucial for ventral cell fate specification. Therefore, our study provides new insights into how a classical factor involved in cell migration also acts on cell fate determination.

### Genetic over-compensation in genetic null mutants

In this study, we faced the genetic compensation responding to gene knockout which was reported recently [33]. Interestingly, the transcription of other MARCKS family members were activated during oogenesis in MZ*marck*sb females, probably driven by non-sense mRNA decay mechanism [53, 54], and Hsp70.3 –the MARCKS interaction protein was up-regulated at shield stage which was presumably driven by zygotic activation in MZ*marcksb* embryos, suggesting a sequential compensation of different genetic factors *via* different mechanisms. Knockdown of either *hsp70*.*3* or a combination of *marcksa*, *marcksl1a* and *marcksl1b* can efficiently block the activity of BMP signaling and reduce the extracellular level of Bmp proteins in the MZ*marcksb* embryos, which indicates that both Hsp70 and other MARCKS proteins collaborate closely to respond to the genetic loss of *marcksb*. In our case, the genetic compensation raised both from genes with sequence homology, and from genes within the same functional network, which support the recently proposed working model [33].

Interestingly, MZ*marcksb* showed a higher level of secreted Bmp2b (Fig 6E and 6F) and was sensitive to the knockdown of Bmp2b antagonist Chordin (Fig 6G–6I), suggesting that the genetic compensation could even lead to elevated output of the overall products and mild enhancement of certain biological process. This phenomenon has never been demonstrated in previous studies. In addition, the detection of maternal expression of other MARCKS family members in MZ*marcksb* suggests that they may have switched from zygotic genes to maternal genes in the genetic adaption process during oogenesis.

## Materials and methods

### Ethics statement

The experiments involving zebrafish followed the Zebrafish Usage Guidelines of the China Zebrafish Resource Center (CZRC) and were performed under the approval of the Institutional Animal Care and Use Committee of the Institute of Hydrobiology, Chinese Academy of Sciences under protocol number IHB2014-006.

### Zebrafish

Embryos were obtained from the natural mating of zebrafish of the AB genetic background (from the China Zebrafish Resource Center, Wuhan, China; Web: http://zfish.cn) and maintained, raised, and staged as previously described [55].

### Constructs, gRNAs and microinjection

For overexpression of proteins, short peptides tags, mCherry or EGFP was inserted in frame after amino acid 295 of Bmp2b according to a previous study [4, 30]. The tagged Bmp2b were inserted into the pCS2+ vector for mRNA synthesis. The constructs of *S4N-marcksb* and *S4D-marcksb* were generated by PCR of the construct of *marcksb-HA* with mutation on the primer pairs. The primer pair for *S4N-marcksb* were F: AACGGTTTCAACTTTAAGAAGAACGCCAAAAAAG and R: CAGCTTGAACGGCTTCTTAAAGTTGAATCG. The primer pair for *S4D-marcksb* were F: GACGGTTTCGACTTTAAGAAGGACGCCAAAAAAGAAG and R: CAGCTTGAACGGCTTCTTAAAGTCGAATCGCTTTTTG (mutated bases in the primer pairs were underlined). Capped mRNA was synthesized using the mMessage mMachine Kit (Ambion). The previously validated morpholino antisense oligonucleotides (MOs) targeting the following genes were used: *marcksa* [39], *marcksb* [25, 39], *chordin* [56], *hsp70*.*3* [38], *marcksl1a* [40], *marcksl1b* [40]. mRNA and MOs were injected into the yolk at the one-cell stage or into one-cell at 32- to 64-cell stage for mosaic injection. Doses for RNAs and MOs were indicated in the text or figures.

### CRISPR/gRNA knock out and generation of mutant fish

The mutants of *marcksb* were generated using CRISPR/Cas9 mediated mutagenesis. The gRNA target for *marcksb* was designed by CRISPRscan [57]. Capped mRNA of zebrafish codon optimized Cas9 [58] and gRNAs of *marcksb* were synthesized by *in vitro* transcription using the mMESSAGE mMACHINE kit (Ambion). 500pg *Cas9* mRNA and 50pg *gRNAs* were co-injected at one-cell stage for each embryo. The gRNA target sequence is as follows: 5’-GGAGCACAAATCTCCAAAAACGG-3’ (the PAM sequence is underlined). The target region was amplified using specific primers of *marcksb* (fwd: 5’-GCGTTGTATCTCGCATCTCAT-3’ and rev 5’-CACACCCCCTCATAACATCA-3’). The PCR products were subject to Sanger sequencing for direct evaluation of the targeting efficiency and identification of mutation [59].

The gRNA targets for *hsp70* (*hsp70*.*1*, *hsp70*.*2* and *hsp70*.*3*), *marcksa*, *marcksl1a* and *marcksl1b* were designed by CRISPRscan [57]. The gRNA target sequences for the above genes were as follows: *hsp70*: 5’-CCTTTAATCCTGAAGAGATTTCC-3’ *marcksa*: 5’-GGCACCGCACCAGCAGAGGATGG-3’; *marcksl1a*: 5’-GGAGAAGCAGTGGCAGCGGACGG-3’; *marcksl1b*: 5’-GGATCCCAGGCATCAAAGGGAGG-3’ (the PAM sequence is underlined). 500pg *Cas9* mRNA and 50pg *gRNAs* were co-injected at one-cell stage for each embryo.

### Whole-mount *in situ* hybridization

Digoxigenin-labeled antisense RNA probes were synthesized by *in vitro* transcription. Whole-mount *in situ* hybridization (WISH) was performed as described [3, 60].

### Cell transplantation

For tail organizer transplantation assay, donor embryos were either injected with *egfp* mRNA or a combination of *egfp* mRNA and *marcksb*_MO at 1-cell stage. Donor embryos were them raised till the shield stage. Approximately 30 donor cells from ventral margin were transplanted to the animal pole of wildtype host embryos of sphere or dome stage as described [28]. Embryos were raised till 1 dpf for evaluation.

### Bmp2b secretion assay

The Bmp2b secretion assay was performed either by mosaic injection or transplantation. For mosaic injection, 50 pg *memGFP* mRNA and 50 pg *mCherry-bmp2b* mRNA with or without 1 ng *marcksb*_MO were injected into one blastoderm cell of a 16-cell to 32-cell stage embryo. The injected embryos were raised till shield stage for confocal imaging.

For transplantation method, 50pg *myc-bmp2b* mRNA and 150 pg *memGFP* mRNA with or without 6 ng *marcksb*_MO were injected into the wildtype fertilized egg. Approximately 30 donor cells at the dome to sphere stage were randomly transplanted into wildtype or *marcksb*-morphant host embryos at the equivalent stage. The correspondent donors and hosts were indicated (Fig 4G-a). Transplanted embryos were screened at shield stage for position identification of donor cells. Embryos were fixed at 60%-epiboly for immunofluorescence staining.

### Immunofluorescence

Immunofluorescence was performed as described [61]. Generally, embryos were fixed in 4% Paraformaldehyde for overnight at 4 ^o^C. Embryos were permeabilized by serial treatments with distilled water for 5 minutes at room temperature, cold acetone for 5 minutes at -20 ^o^C, distilled water for 5 minutes at room temperature. For immunofluorescence of P-Smad1/5/9, all the steps before adding secondary antibody should be performed under 4 ^o^C. Anti-Phospho-Smad1/5/9 (D5B10) Rabbit mAb (CST) was used at dilution 1:500. Anti-Myc (Santa Cruz) was used at dilution 1:500. Anti-rabbit Alexa Fluor 568 were used as secondary antibody (Molecular probes) at dilution 1:500. Embryos were counterstained with DAPI (5mg/ml in stock, 1:5000 diluted with PBS for working solution) for 1 hour. After immunofluorescence, the embryos were kept in 50% glycerol-50%PBS with 1mg/ml anti-fade reagent phenylenediamine (Sigma) avoid from light at 4 ^o^C.

### Microscopy and imaging processing

Confocal images were acquired using a laser-scanning confocal inverted microscope (SP8, Leica) with a LD C-Apo 40×/NA 1.1 water objective. Z-stacks were generated from images taken at 0.5 μm intervals, using the following settings (2048x2048 pixel, 400MHz). For detection of p-Smad1/5/9 signal, confocal images were acquired by the same scope using Lan-Apo 20x/NA 0.75 objective at zoom 0.75. Z-stacks were generated from images taken at 3 μm intervals, using the following settings (1024x1024 pixel, 400MHz). Embryos of shield to 60% epiboly stage were mounted in 0.5% low-melting agarose and positioned with animal pole to the bottom.

The Fiji software was used to quantify the average fluorescent intensity of P-Smad and the secreted Bmp2b protein [62].

For quantifying the P-Smad1/5/9 intensity, all the embryos were firstly orientated as dorsal region to the right. 8-bit image of each channel was transformed into 32-bit image. The threshold was made using default method and the background was set to NaN. A rectangle selection tool was used to select an area covering the whole embryo. The selected area was added to the ROI manager. The intensity from ventral to dorsal region of both P-Smad1/5/9 and DAPI were measure by plot profile function of Fiji. The data were then exported into Microsoft Excel and calculated. The ratio of P-Smad to DAPI from ventral region to dorsal region was plotted using GraphPad Prism 7.

For quantifying the secreted mCherry-Bmp2b or myc-Bmp2b, the 8-bit image was first transformed into 32-bit. The threshold was made using default method and the background was set to NaN. A polygon selection tool was used to select an area covering the outside of Bmp2b-source cell. A total selected area (Area_total_) and the area (Area_threshold_) limited to the threshold were measured. The secreted Bmp2b (Bmp2b_secreted_) was calculated by the formula: Bmp2b_secreted_ = Area_threshold_ / Area_total._ The data was plotted using GraphPad Prism 7 as scatterplots with median for small sample size studies [63].

### Immunoprecipitation and western blot

Co-immunoprecipitation experiments were performed as described previously [64]. For immunoprecipitation assays, the cDNAs of *marcksa*, *marcksb*, *marcksl1a* and *marcksl1b* were cloned into pCS2+MTC (C-terminal multiple myc tag) and hsp70.3 was cloned into pCGN-HAM (N-terminal multiple HA tag) vectors. HEK293T cells were transiently transfected with the indicated constructs of interest using VigoFect (Vigorous Biotechnology, China) at dosage of 10 μg plasmid for cells covering about 70% surface of culture bottle (Nest, 100mm cell culture Dish).

For immunoblotting of intracellular and extracellular Bmp2b in cultured 293T cells, 10 μg of endotoxin-free pCS2-myc-bmp2b or pCS2-mcherry-bmp2b were transfected for cells covering 70% surface of culture bottle. After 8 hours, we replaced the growth medium (DMEM (high glucose, Biological Industries, 01-052-1ACS) with 10% FBS (Biological Industries, 04-001-1A)) with serum-free high-glucose DMEM and cultured cells for another 12 hours. One bottle of cells and growth medium were collected separately. Cells was lysed with 500μl RIPA buffer (50 mM Tris at pH 7.4, 150 mM NaCl, 1% NP-40, 0.5% deoxycholate, 1 mM NaF, 1 mM EDTA and protease inhibitors) at 4 ^o^C. The protein concentration was measured by Enhanced BCA Protein Assay Kit (Beyotime Biotechnology, P0010). About 50 μg protein was loaded to a lane for immunoblotting. The growth medium was centrifuged at 300g for several minutes to precipitate the cells and the supernatant was collected and concentrated by Centrifugal ultrafiltration tube (Amicon Ultra UFC9001096 and UFC5010BK).

For immunoblotting of embryonic and extracellular Bmp2b *in vivo*, zebrafish embryos were either injected with 10 pg *mCherry-bmp2b* mRNA per embryo or co-injected with 10 pg *mCherry-bmp2b* mRNA and 6 ng *marcksb*_MO per embryo. Each of 300 embryos at shield stage were harvested and dissociated by pipetting in 350 μL calcium-free Ringer’s solution. The cells were collected by centrifugation at 300 g for several minutes. The cells were then lysed with RIPA, vortexed vigorously, added with 5xSDS loading buffer (Beyotime Biotechnology, P0015), incubated for 10 minutes at 95 ^o^C and used for immunoblotting. 5 embryos were loaded for each lane. 300 μL supernatant was incubated with mouse anti-mCherry antibody (Abclonal, AE002) embedded Protein G beads (Life, Dynabeads protein G, 10003D) (10 μL antibody for 50 μL beads) overnight at 4 ^o^C. After washing 3 times with PBS with 0.02% Tween-20, the beads were added with RIPA and 5xSDS loading buffer, incubated for 10 minutes at 95 ^o^C and used for immunoblotting.

For immunoblotting, anti-Myc (Santa Cruz Biotechnology, 1:2000), anti-HA (Sigma-Aldrich, 1:5000), anti-mCherry (Abclonal, AE002) antibodies were used.

### RNA sequencing (RNA-Seq) and analysis

Two hundred Embryos of either wildtype or MZ*marcksb* at shield stage were divided into two groups as replicates. The RNA was extracted using Trizol according to the manufacturer’s manual. Then the RNA was purified using RNA purification kit (Tiangen, China). The RNA samples were quantified and integrity was assessed by the Agilent 2100 Bioanalyser. The RNA integrity Numbers (RIN) of all RNA samples were >8.0. The RNA libraries were prepared using the Illumina TruSeq RNA sample preparation kit v2. The amount of input RNA is 1 μg. The average final library size is 309 bp. Sequencing was performed on Illumina Miseq with read length of 150 bp paired-end (PE) at the Analysis and Testing Center of Institute of Hydrobiology, Chinese Academy of Sciences. Clean data were mapped to zebrafish reference genome GRCz10 Ensembl release 87 using HISAT2 with default parameters [65]. Cuffquant and Cuffnorm from Cufflinks software package were used to calculate the normalized gene expression level [66, 67]. The differential expression analysis was performed using DEseq2 [68]. The original RNA-seq data has been deposited to the BioProject with accession number PRJNA432757 (https://www.ncbi.nlm.nih.gov/bioproject/PRJNA432757).

### Reverse-transcription quantitative PCR (RT-qPCR) assay

Wildtype, MZ*marcksb* and M*marcksb* embryos at 1-cell stage and shield stage, and *marcksb* morphants at shield stage were collected for RNA extraction and reverse-transcription with about 60~70 embryos per sample and at least biological triplicate. The BioRad CFX Connect Real-Time System was used for transcript quantification. Samples were tested in technical triplicate for each gene, and resultant Cq values were averaged. Primer efficiencies and gene expression levels were calculated according to the previous study [69]. *eef1a* was selected as reference gene. Data were processed using 2^-ΔΔCq^ method. All RT-qPCR gene-specific primers are listed in S1 Table.

## Supporting information

S1 Fig Whole-mount *in situ* analysis of four MARCKS members during early embryogenesis. (A-D) WISH analysis of *marcksa*. (E-H) WISH analysis of *marcksb*. (I-L) WISH analysis of *marcksl1a*. (M-P) WISH analysis of *marcksl1b*. (A, E, I, M) Embryos are at 2-cell stage, lateral view with animal-pole to the top; (B, F, J, N) Embryos are at high stage, lateral view with animal-pole to the top; (C, G, K, O) Embryos are at shield stage, lateral view with animal-pole to the top and dorsal to the right; (D, H, L, P) Embryos are at 75%-epiboly stage, lateral view with animal-pole to the top and dorsal to the right. *marcksb* shows strong maternal expression and its high expression level lasts to 75%-epiboly stage; there is no maternal transcription of *marcksa*, *marcksl1a*, *marcksl1b* and their zygotic transcripts could only be detected from shield stage.
(TIF)

S2 Fig Knockdown of *marcksb* did not cause dorsalization in MZ*marcksb* or M*marcksb*. (A-D) WISH of dorsal marker *otx2* (neural ectoderm). (E-H) WISH of ventral marker *foxi1* (non-neural ectoderm). (I, J) WISH of BMP signaling target *szl*. (K, L) WISH of BMP signaling target *ved*. (A, E) maternal-zygotic mutant of *marcksb* (MZ*marcksb*). (B, F) MZ*marcksb* injected with 6 ng of *marcksb*_MO (MZ*marcksb+ marcksb*_MO). (C, G, I, K) maternal-only mutant of *marcksb* (M*marcksb*). (D, H, J, L) M*marcksb* injected with 6 ng of *marcksb*_MO (M*marcksb+ marcksb*_MO).For *otx2*, *szl* and *ved*, the representative embryos were animal view with dorsal to the right. For *foxi1*, the representative embryos were lateral view with animal-pole to the top and dorsal to the right. The developmental stages of embryos were indicated in the figure. The number of embryos with representative phenotype slash total embryo number was indicated at the lower right corner of each image.
(TIF)

S3 Fig Knockdown of either *hsp70* or three MARCKS genes (*marcksa*, *marcksl1a* and *marcksl1b*) by moderate dosage of morpholino injection in wildtype embryos mildly decreased the expression of *szl*. (A) The expression of *szl* was mildly decreased in wildtype embryos injected with either *hsp70*_MO or *marcksa_l1a_l1b*_MOs. The embryos are at shield stage and lateral view with dorsal to the right. (B) The percentage of embryos with normal-like and mildly decreased expression of *szl*. “n” represents the number of embryos we observed.
(TIF)

S4 Fig Validation of gRNAs by sequencing of the target sites. (A) Sequence data from *hsp70*_gRNA injected embryos covering the target site; (B) Sequence data from *marcksa*_gRNA injected embryos covering the target site;(C) Sequence data from *marcksl1a*_gRNA injected embryos covering the target site; (D) Sequence data from *marcksl1b*_gRNA injected embryos covering the target site. The targets sites were shown in black box. Sequences of Protospacer adjacent motif (PAM) were red-underlined. Please note that the place where multi-peaks at each nucleotide position began revealed the starting point of mutation.
(TIF)

S5 Fig The BMP signaling activity was decreased either by knockdown of *hsp70.3* alone or by simultaneous knockdown of *marcksa*, *marcksl1a* and *marcksl1b* using CRISPR/Cas9 mediated approach. (A-B) WISH analysis of *szl* (A) and *ved* (B). The percentage of embryos with different phenotypes for each group indicated in the graph; embryos of shield stage are animal-pole view with dorsal to the right; “n” represents the number of embryos we observed.
(TIF)

S6 Fig Knockdown of *hsp70* with full dosage of *hsp70*_MO led to decreased BMP signaling activity. (A-C) WISH analysis of BMP signaling target *szl*; (D) The percentage of embryos with normal-like, decreased and rescued phenotypes shown by *szl* expression. “n” represents the number of embryos we observed. (E-G) WISH of BMP signaling target *ved*; (A, E) Wildtype embryos; (B, F) Wildtype embryos injected with 6ng of *hsp70*_MO (*hsp70*_MO); (C, G) *Hsp70* morphants injected with 150 pg of morpholino-insensitive *hsp70*.*3* mRNA; (H) The percentage of embryos with normal-like, decreased and rescued phenotypes shown by *ved* expression. “n” represents the number of embryos we observed. Embryos of shield stage are animal-pole view with dorsal to the right.
(TIF)

S1 Table RT-qPCR gene-specific primers used in this study. (DOCX)

S1 Dataset Differential expression gene list between wildtype and MZ*marcksb* at shield stage. (XLSX)

S1 File Numerical data. This file contains statistical data corresponding to all graphs presented in the manuscript.
(ZIP)
