# Supplementary figures and images for: Marcksb plays a key role in the secretory pathway of zebrafish Bmp2b
Source: PLoS Genet. 2019 Sep 23;15(9):e1008306. doi: 10.1371/journal.pgen.1008306 (PMC6776368; doi:10.1371/journal.pgen.1008306)

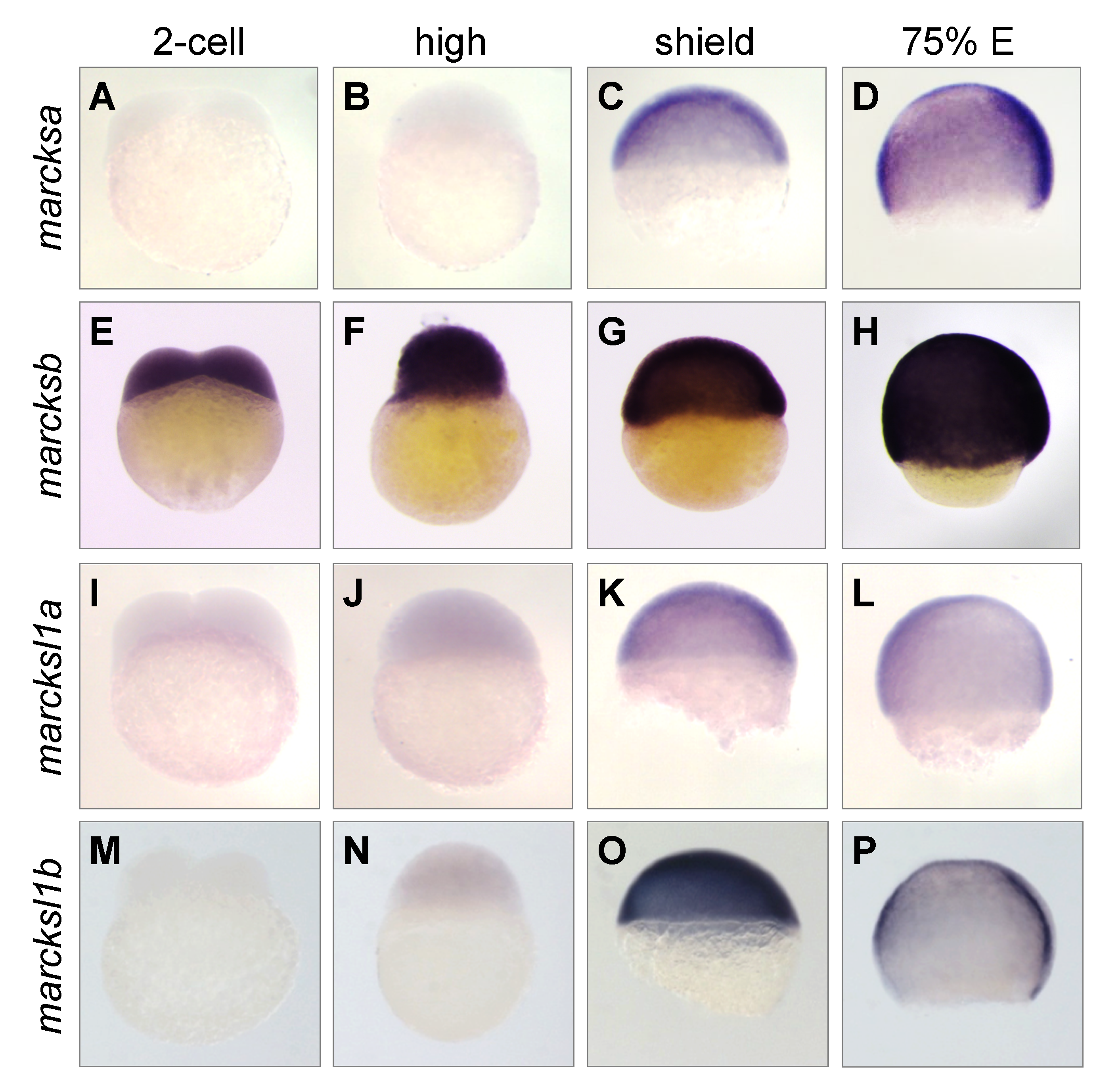

Supplement: S1 Fig — (A-D) WISH analysis of marcksa. (E-H) WISH analysis of marcksb. (I-L) WISH analysis of marcksl1a. (M-P) WISH analysis of marcksl1b. (A, E, I, M) Embryos are at 2-cell stage, lateral view with animal-pole to the top; (B, F, J, N) Embryos are at high stage, lateral view with animal-pole to the top; (C, G, K, O) Embryos are at shield stage, lateral view with animal-pole to the top and dorsal to the right; (D, H, L, P) Embryos are at 75%-epiboly stage, lateral view with animal-pole to the top and dorsal to the right. marcksb shows strong maternal expression and its high expression level lasts to 75%-epiboly stage; there is no maternal transcription of marcksa, marcksl1a, marcksl1b and their zygotic transcripts could only be detected from shield stage. (TIF) [file pgen.1008306.s001.tif]

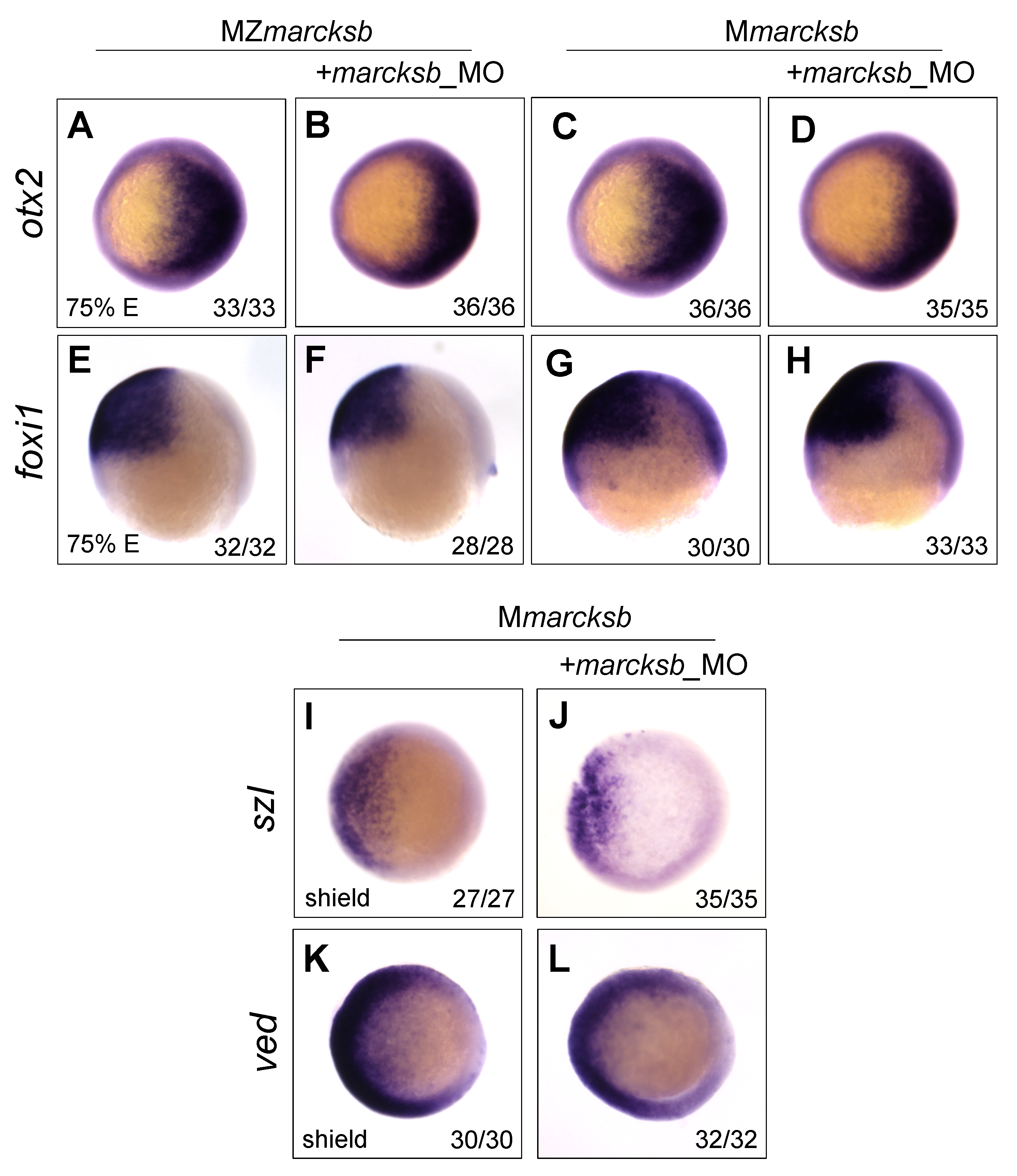

Supplement: S2 Fig — (A-D) WISH of dorsal marker otx2 (neural ectoderm). (E-H) WISH of ventral marker foxi1 (non-neural ectoderm). (I, J) WISH of BMP signaling target szl. (K, L) WISH of BMP signaling target ved. (A, E) maternal-zygotic mutant of marcksb (MZmarcksb). (B, F) MZmarcksb injected with 6 ng of marcksb_MO (MZmarcksb+ marcksb_MO). (C, G, I, K) maternal-only mutant of marcksb (Mmarcksb). (D, H, J, L) Mmarcksb injected with 6 ng of marcksb_MO (Mmarcksb+ marcksb_MO).For otx2, szl and ved, the representative embryos were animal view with dorsal to the right. For foxi1, the representative embryos were lateral view with animal-pole to the top and dorsal to the right. The developmental stages of embryos were indicated in the figure. The number of embryos with representative phenotype slash total embryo number was indicated at the lower right corner of each image. (TIF) [file pgen.1008306.s002.tif]

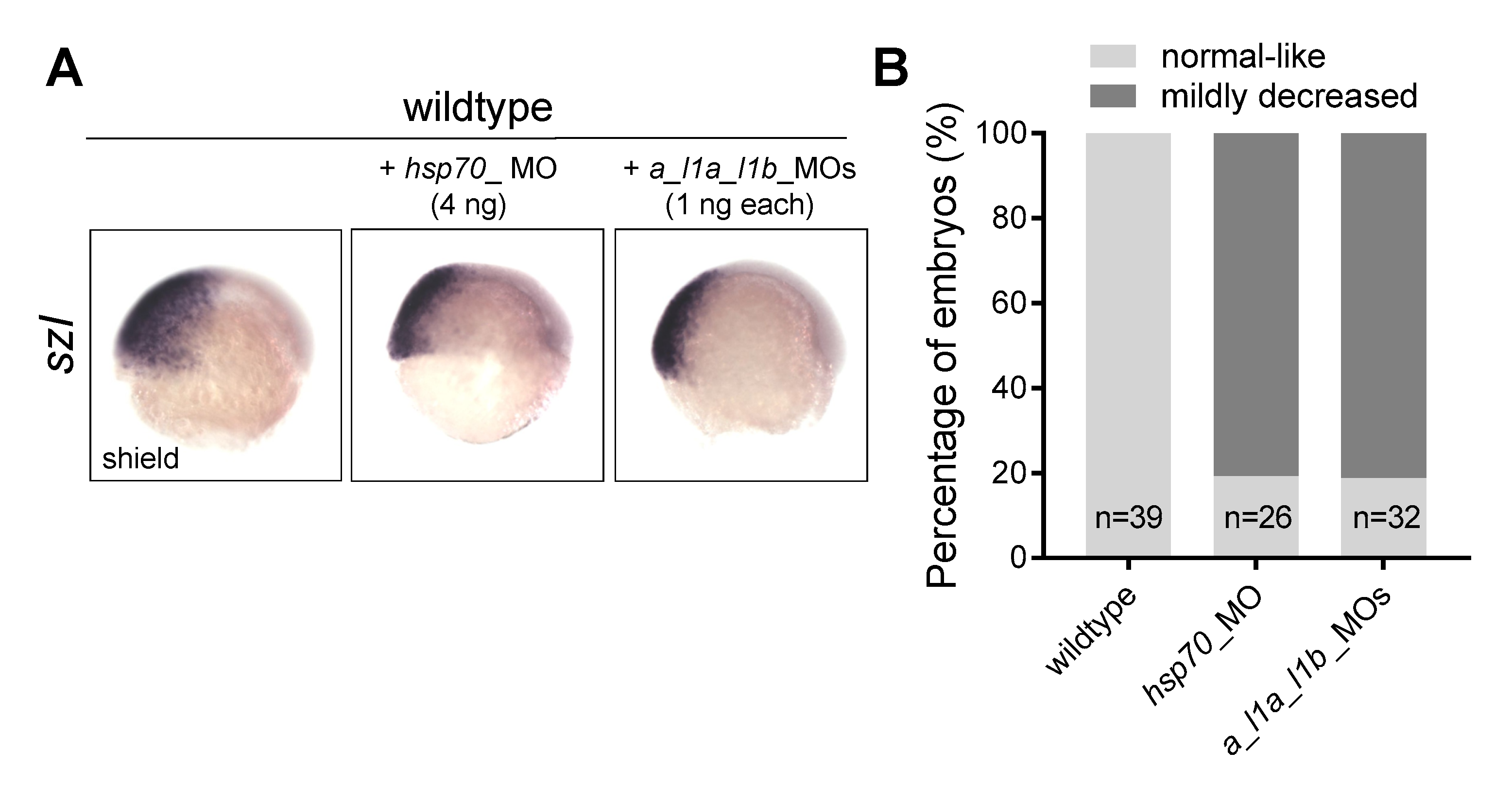

Supplement: S3 Fig — (A) The expression of szl was mildly decreased in wildtype embryos injected with either hsp70_MO or marcksa_l1a_l1b_MOs. The embryos are at shield stage and lateral view with dorsal to the right. (B) The percentage of embryos with normal-like and mildly decreased expression of szl. “n” represents the number of embryos we observed. (TIF) [file pgen.1008306.s003.tif]

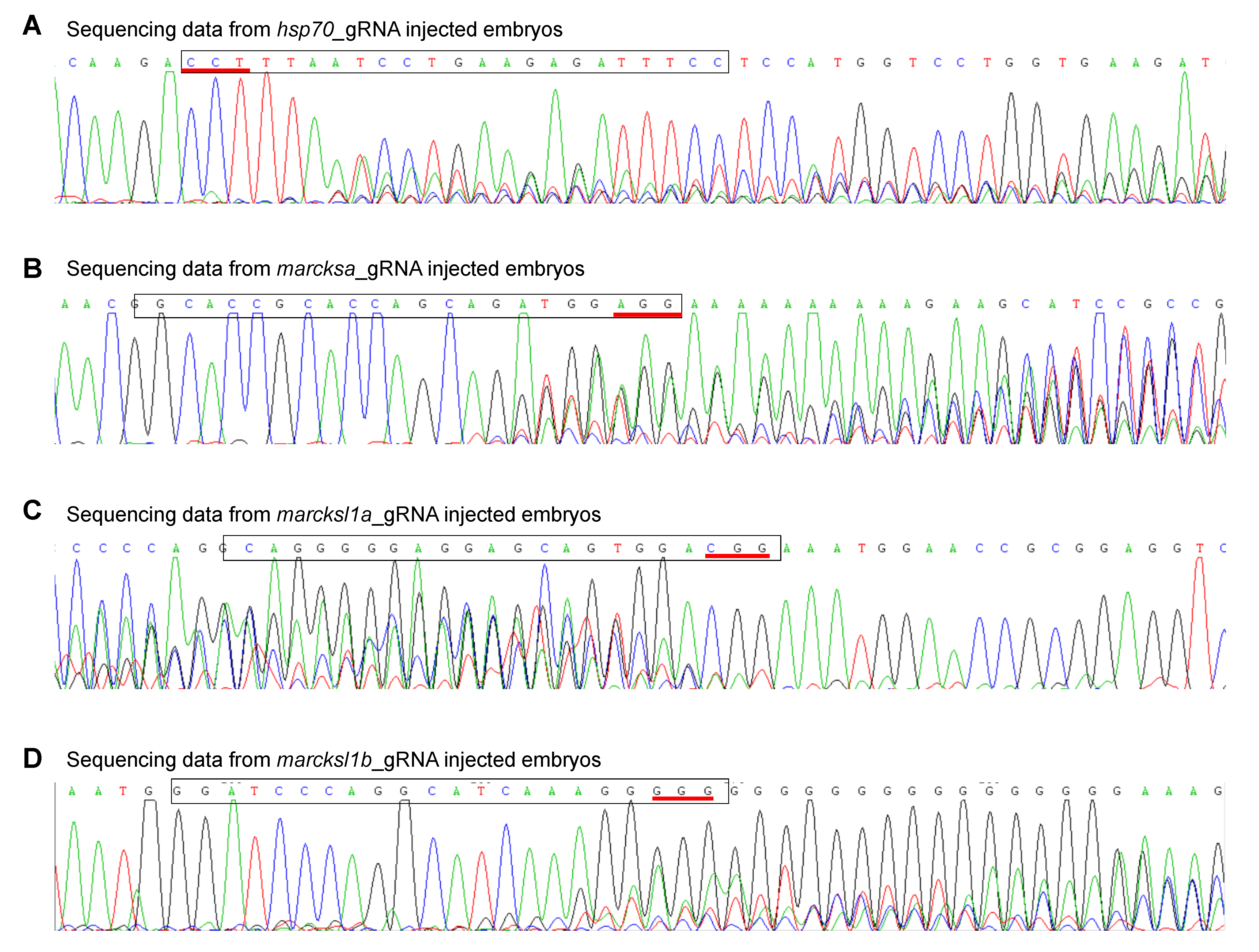

Supplement: S4 Fig — (A) Sequence data from hsp70_gRNA injected embryos covering the target site; (B) Sequence data from marcksa_gRNA injected embryos covering the target site;(C) Sequence data from marcksl1a_gRNA injected embryos covering the target site; (D) Sequence data from marcksl1b_gRNA injected embryos covering the target site. The targets sites were shown in black box. Sequences of Protospacer adjacent motif (PAM) were red-underlined. Please note that the place where multi-peaks at each nucleotide position began revealed the starting point of mutation. (TIF) [file pgen.1008306.s004.tif]

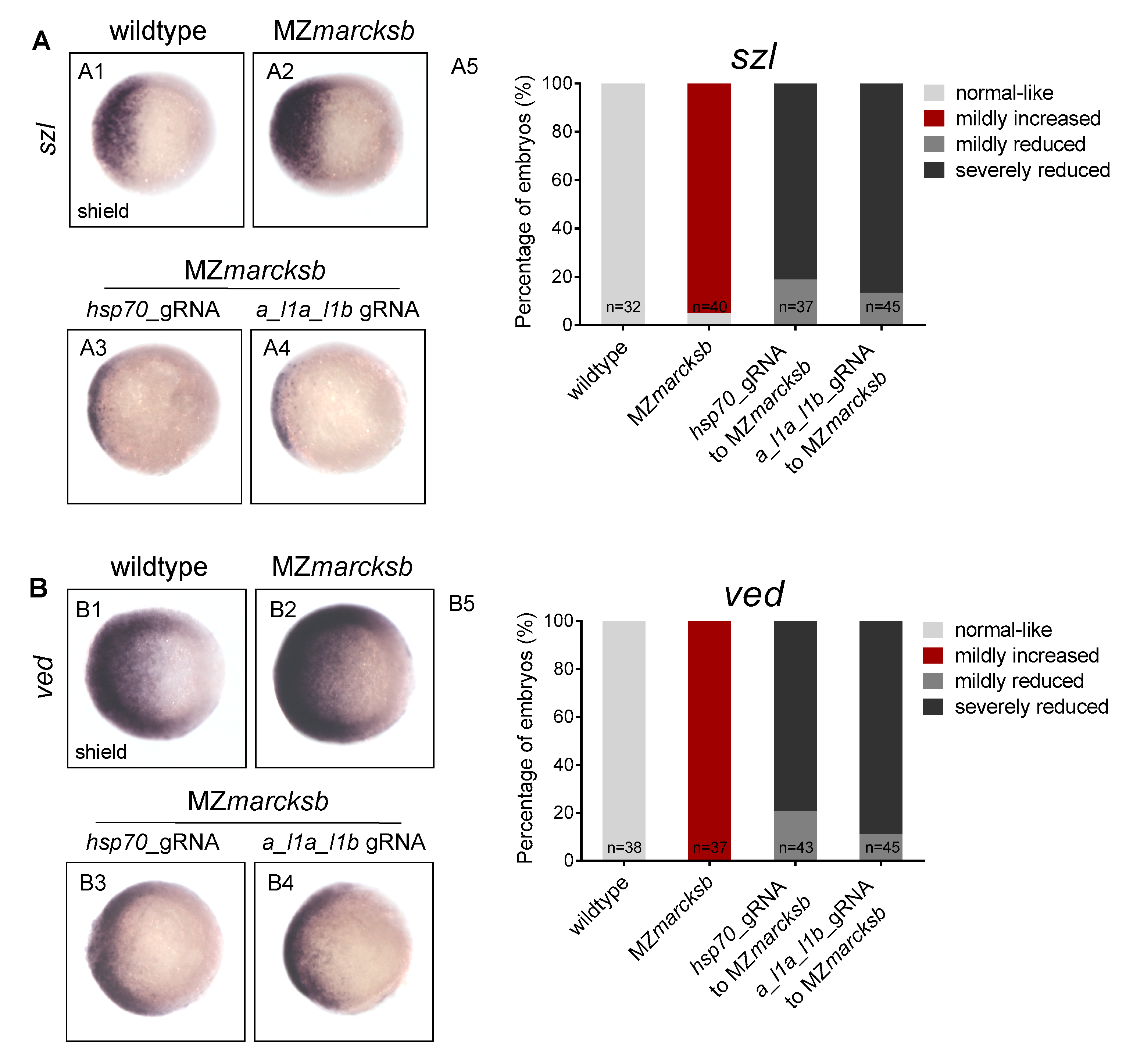

Supplement: S5 Fig — (A-B) WISH analysis of szl (A) and ved (B). The percentage of embryos with different phenotypes for each group indicated in the graph; embryos of shield stage are animal-pole view with dorsal to the right; “n” represents the number of embryos we observed. (TIF) [file pgen.1008306.s005.tif]

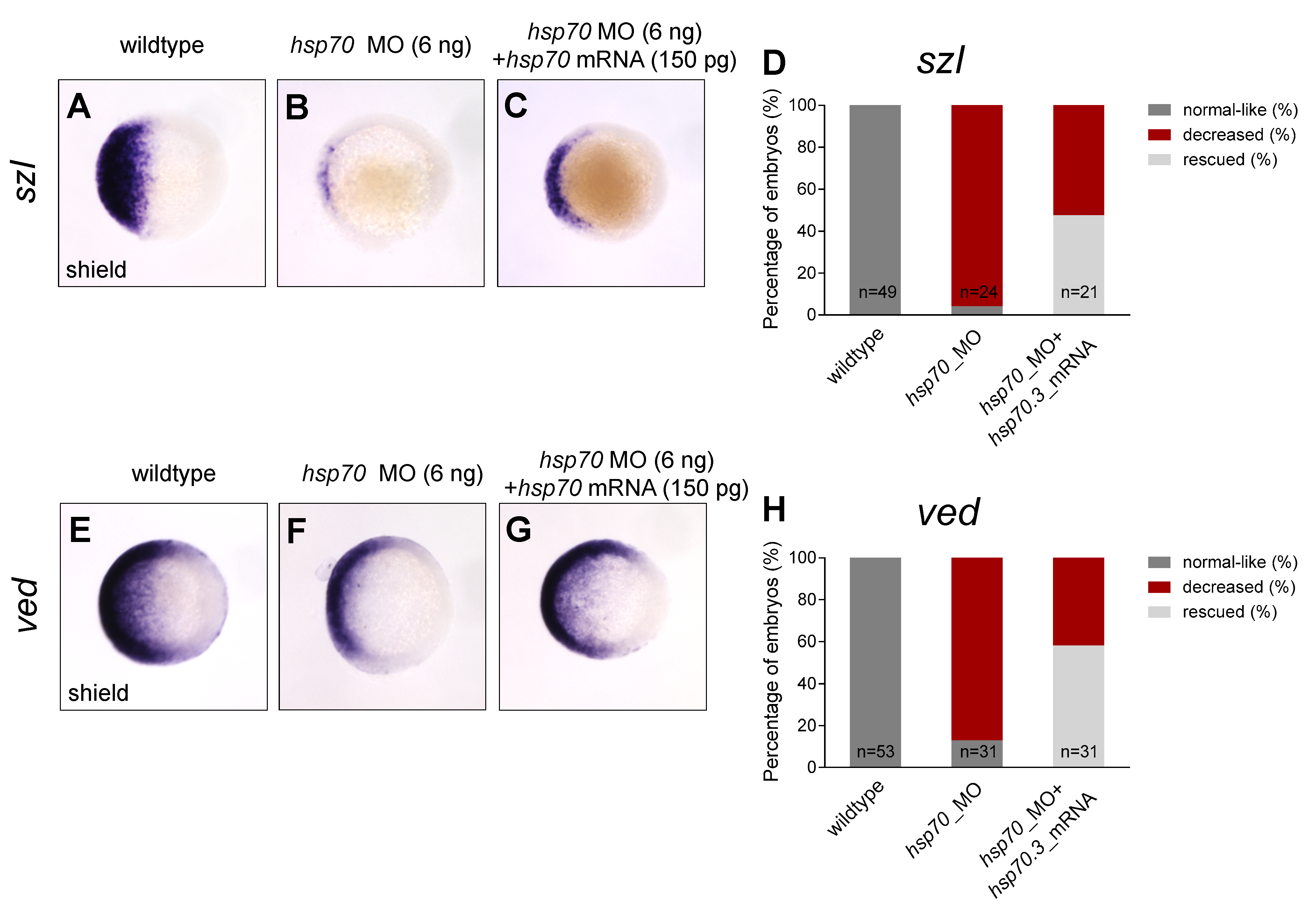

Supplement: S6 Fig — (A-C) WISH analysis of BMP signaling target szl; (D) The percentage of embryos with normal-like, decreased and rescued phenotypes shown by szl expression. “n” represents the number of embryos we observed. (E-G) WISH of BMP signaling target ved; (A, E) Wildtype embryos; (B, F) Wildtype embryos injected with 6ng of hsp70_MO (hsp70_MO); (C, G) Hsp70 morphants injected with 150 pg of morpholino-insensitive hsp70.3 mRNA; (H) The percentage of embryos with normal-like, decreased and rescued phenotypes shown by ved expression. “n” represents the number of embryos we observed. Embryos of shield stage are animal-pole view with dorsal to the right. (TIF) [file pgen.1008306.s006.tif]
