# Supplementary material for: Marcksb plays a key role in the secretory pathway of zebrafish Bmp2b
Source: PLoS Genet. 2019 Sep 23;15(9):e1008306. doi: 10.1371/journal.pgen.1008306 (PMC6776368; doi:10.1371/journal.pgen.1008306)
Supplement: S1 Table — (DOCX) [file pgen.1008306.s007.docx]

**Supplementary Table S8. RT-qPCR gene-specific primers used in this study**

| *marcksa*  Fwd: ATACGGAAAATGGCCACCTG  Rev: ATCGCTTCTGTGTTTCCATCTG |
| --- |
| *marcksb*  Fwd: CACGCGACGCGTCTATGA  Rev: GAGAGGCATTCCCGTTGGT |
| *marcksl1a*  Fwd: CTCTCGCTCACCGCATTTACT  Rev: CTTGGTGGATACATCTCCGTTAGT |
| *marcksl1b*  Fwd: CGGCTGCCGTCAAGACTAA  Rev: CTCCAGCCTCAGATTCCTTAGC |
| *hsp70.3*  Fwd: GGAACACAAAGGAGAAAACAAGAC  Rev: TGGGAGTCGTTGAAATAGGCT |
